# Translational Aspects of DNA Damage Repair in Optimizing Cancer Chemotherapy

**DOI:** 10.1002/ggn2.202500036

**Published:** 2025-12-22

**Authors:** Anqi Lin, Jinyue He, Aimin Jiang, Jian Zhang, Quan Cheng, Hengguo Zhang, Wenjun Mao, Peng Luo

**Affiliations:** ^1^ Department of Oncology Zhujiang Hospital Southern Medical University Guangzhou China; ^2^ Department of Urology Changhai Hospital Naval Medical University (Second Military Medical University) Shanghai China; ^3^ Department of Neurosurgery Xiangya Hospital Central South University Changsha Hunan China; ^4^ National Clinical Research Center for Geriatric Disorders Xiangya Hospital Central South University Hunan China; ^5^ College & Hospital of Stomatology Key Lab. of Oral Diseases Research of Anhui Province Anhui Medical University Hefei China; ^6^ Department of Thoracic Surgery Wuxi People's Hospital Wuxi Medical Center The Affiliated Wuxi People's Hospital of Nanjing Medical University Nanjing Medical University Wuxi China

**Keywords:** DNA damage repair, chemotherapy, personalized treatment, drug resistance, DDR inhibitors

## Abstract

The DNA Damage Repair (DDR) signaling pathway serves as a crucial molecular hub that regulates chemotherapy efficacy, offering significant translational value in the field of precision oncology. This review systematically analyzes the molecular mechanisms of five core DDR pathways (Homologous Recombination Repair, Non‐Homologous End Joining, Base Excision Repair, Nucleotide Excision Repair, and Mismatch Repair) in mediating chemotherapy resistance in tumors, and thoroughly elucidates the correlation between key molecular events—such as BRCA1/2 deficiency, MMR functional abnormalities, and Ataxia Telangiectasia Mutated/Ataxia Telangiectasia and Rad3‐related (ATM/ATR) signaling pathway dysregulation—and chemotherapy sensitivity. The DDR deficiency biomarker system established through the integration of multi‐omics data provides molecular classification tools for predicting the efficacy of platinum‐based drugs. This study focuses on the mechanism by which Poly ADP‐Ribose Polymerase inhibitors reverse Homologous Recombination‐Deficient tumor resistance through “synthetic lethality” effects while also revealing the synergistic anti‐tumor effects of ATM/ATR inhibitors in combination with chemotherapeutic agents. The research presents an innovative molecular synergy model between DDR regulation and Immune Checkpoint Blockade, confirming that tumor neoantigen release induced by DDR deficiency can enhance immunotherapy responses. This article also provides perspectives on multidimensional intervention strategies based on the DDR network, including the development of inhibitors targeting novel DDR targets, the establishment of DDR pathway functional assessment systems based on multidimensional biomarkers, and the investigation of synergistic paradigms between DDR and novel therapeutic modalities. Additionally, we explore the dynamic evolution mechanisms of DDR‐mediated chemotherapy resistance by analyzing the interactions between DDR and metabolic reprogramming, as well as other related processes. These breakthrough advances provide theoretical foundations and innovative directions for overcoming chemotherapy resistance and advancing personalized treatment, marking a new era in cancer therapy characterized by precision targeting of DDR pathways.

## Graphical abstract

This review systematically delineates the fundamental role and recent advancements in DNA damage response (DDR) within the context of cancer chemotherapy. Particular emphasis is directed toward exploring the interplay between DDR status and chemosensitivity. Furthermore, prospective directions and emerging challenges in this field are critically discussed.

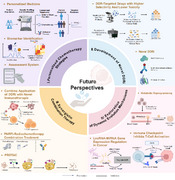

**Abbreviations**
ATM: Ataxia Telangiectasia Mutated
ATR: Ataxia telangiectasia and Rad3‐related
BER: Base Excision Repair
CHK1/2: Checkpoint Kinase1/2
CircRNA: Circular RNA
CSCs: cancer stem cells
DDR: DNA Damage Repair
DDRi: DNA Damage Response inhibitors
DNA‐PK: DNA‐dependent protein kinase
DNA‐PKcs: DNA‐dependent protein kinase catalytic subunit
DSBs: Double‐Strand Breaks
ESC: embryonic stem cell
HR: Homologous Recombination
HRD: Homologous Recombination deficient
HRR: Homologous Recombination Repair
HRRm: HRR gene mutation
ICB: immune checkpoint blockade
ICLs: inter‐strand crosslinks
LncRNAs: Long Non‐Coding RNAs
MiRNAs: MicroRNAs
MMR: Mismatch Repair
MSI: microsatellite instability
NER: Nucleotide Excision Repair
NHEJ: Non‐Homologous End Joining
NSCLC: non‐small cell lung cancer
PARP: Poly ADP‐ribose polymerase
PARPi: PARP inhibitors
PGE2: Prostaglandin E2
PROTAC: Proteolysis Targeting Chimera
PTM: Post‐translational modification
SSBs: Single Strand Breaks
TMB: Tumor Mutational Burden
TME: Tumor Microenvironment

## 1 Introduction

The human cellular genome, when continuously exposed to various environmental factors, is susceptible to DNA damage and replication abnormalities. The DNA Damage Repair (DDR) is a complex regulatory system that evolved to maintain genomic stability and respond to DNA damage caused by both exogenous and endogenous factors [1, 2]. This system encompasses various DNA repair mechanisms, damage tolerance pathways, and cell cycle checkpoints, thereby playing a crucial role in the occurrence, progression, and treatment of tumors [3]. The DDR network recognizes multiple types of DNA damage, thereby activating cell cycle regulatory mechanisms and recruiting DNA repair factors to acilitate repair of the damaged genome. The DDR system plays a critical role in maintaining genetic integrity and suppressing mutation generation; however, this process may paradoxically contribute to tumorigenesis [4]. In the precancerous stage in humans, activated oncogenes and inactivated tumor suppressor proteins trigger DDR pathways through induced replication stress, enhancing DNA repair and establishing an anti‐cancer barrier. However, this selective pressure may lead to mutations or epigenetic silencing of DDR genes, ultimately facilitating tumorigenesis [5]. During malignant tumor progression, high levels of DNA replication stress resulting from rapid proliferation of tumor cells cannot be repaired promptly due to DDR functional deficiencies, thereby exacerbating genomic instability and accelerating tumor progression [6].

In clinical applications, the anti‐tumor activity of chemotherapeutic agents is closely correlated with their mechanisms of inducing DNA damage [7, 8, 9, 10, 11], which can be classified into several categories: alkylating agents that induce DNA Single‐Strand Breaks (SSBs) and Double‐Strand Breaks (DSBs), thereby inhibiting normal cell division and proliferation [8]; antibiotic drugs intercalate into DNA double‐strand structures, impeding the normal function of DNA polymerases; platinum‐based chemotherapeutic agents exert their anti‐tumor effects by forming DNA adducts and DNA Interstrand Cross‐Links (ICLs) [12]. Chemotherapeutic drugs exert anti‐tumor effects by inducing DNA damage; however, activation of the DDR mechanism may attenuate the therapeutic efficacy of these drugs. DDR exhibits a dual role in tumor occurrence, development, and treatment: on one hand, during the normal cell cycle, the timely repair of DNA damage is indispensable for maintaining genomic stability, thereby averting cellular mutations and tumorigenesis [13]; conversely, the DDR system may facilitate the emergence of chemoresistance by repairing DNA lesions induced by chemotherapeutic agents [14]. Evidence suggests that the survival of damaged cells correlates with germline functional alterations in DDR genes; mechanisms including epigenetic silencing and tumor microenvironment modulation enhance DDR pathway activity, thereby repurposing the protective role of DDR into a critical driver of tumor progression that enables cancer cell tolerance to DNA damage and adaptation to chemotherapy [15]. Research demonstrates that tumor cell sensitivity to chemotherapeutic agents is negatively correlated with their DDR capacity [16]. Abnormal activation of DNA repair mechanisms is one of the key mechanisms underlying chemotherapy resistance in tumor cells [17]. DDR, by repairing treatment‐induced DNA damage, can diminish tumor cell sensitivity to anticancer DNA‐damaging chemotherapeutic agents, thereby facilitating tumor progression and fostering the development of resistance [18].

DNA repair pathways are widely recognized as promising targets in clinical chemotherapy. DDR‐targeted therapy inhibits DDR pathways, thus preventing cancer cells from effectively repairing DNA damaged by chemotherapeutic agents and subsequently inducing cell apoptosis. For instance, Ataxia Telangiectasia Mutated (ATM) inhibitors can suppress the expression of ATM protein in cells, thereby compromising DDR efficacy; Poly ADP‐Ribose Polymerase (PARP) inhibitors (PARPi) block the expression of PARP enzymes involved in DDR, resulting in DNA damage accumulation and ultimately triggering cancer cell apoptosis [19]. Concurrently, the combination of DDR inhibition with other anticancer therapies demonstrates significant potential, and the interaction between chemotherapy resistance and DDR inhibitors (DDRi) represents an attractive research area in cancer treatment. For example, the combination of Ataxia Telangiectasia and Rad3‐related (ATR) inhibitors with cisplatin can significantly overcome cisplatin resistance [20]. The combination of chemotherapeutic agents with DDRi has improved chemotherapy outcomes, thus potentially offering a novel strategy to overcome clinical tumor recurrence [21]. Furthermore, the combination of DDR inhibition with immunotherapy continues to evolve, emerging as an effective strategy to enhance anti‐tumor effects. With further research, the relationship between DDR and chemotherapy will be elucidated more comprehensively, yielding significant benefits for cancer patients.

Despite significant progress in DDR research in the field of chemotherapy, numerous critical challenges remain to be resolved. The potential mechanisms of DDR dysregulation in cancer and their impact on clinical chemotherapy largely remain elusive, and both the understanding and application of DDR‐targeted therapy are not yet sufficiently comprehensive and specific [22]. Additionally, clinically available DDR combination therapy regimens frequently exhibit significant toxicity [23]. Personalized treatment requires more comprehensive analysis and integration of tumor DDR status during chemotherapy to achieve enhanced diagnostic resolution. This review systematically examines the latest research progress of DDR in tumor chemotherapy and its clinical application prospects. First, this article thoroughly analyzes the key molecular mechanisms of the five major DDR pathways in maintaining genomic stability and mediating the formation of chemotherapy resistance. Second, it focuses on the correlation between DDR and chemotherapy sensitivity, systematically elucidating the molecular mechanisms by which functional defects in key DDR‐related genes affect tumor cell sensitivity to chemotherapeutic agents by regulating DNA repair capabilities. Third, it comprehensively summarizes chemotherapy sensitization strategies targeting DDR, systematically assessing the clinical application value of various DDRi in overcoming chemotherapy resistance. Fourth, it discusses the molecular‐level synergistic mechanisms that exist between DDR pathways and immunotherapy. Finally, this article provides perspectives on future directions in this field, including the development of personalized chemotherapy strategies targeting DDR pathways and the advancement of novel DDRi to overcome chemotherapy resistance. Based on the current research status, further advancing the combined treatment paradigm of DDR‐chemotherapy synergy, investigating the regulatory network between DDR signal transduction networks and non‐coding RNAs, and exploring the dynamic evolution mechanisms of DDR‐mediated chemotherapy resistance will provide novel therapeutic approaches and research directions for advancing precision oncology.

## 2 Major DDR Pathways and Their Roles in Chemotherapy

DDR, as a key mechanism for maintaining genomic stability, plays a crucial role in tumor initiation, progression, and the development of chemotherapy resistance. The DDR system primarily consists of repair pathways including Homologous Recombination Repair (HRR), Non‐Homologous End Joining (NHEJ), Base Excision Repair (BER), Nucleotide Excision Repair (NER), and Mismatch Repair (MMR). These repair mechanisms recognize and repair different types of DNA damage through specific molecular pathways, exhibiting dual effects in response to chemotherapy‐induced DNA damage: they maintain genomic stability in normal cells while potentially mediating the development of therapeutic resistance in tumor cells. In‐depth elucidation of DDR pathway regulatory networks and their mechanisms of action during chemotherapy provides significant theoretical and practical implications for developing novel antitumor drugs and optimizing clinical treatment strategies.

### 2.1 HRR

HRR is a key mechanism for repairing DSBs during the S and G2 phases of the cell cycle, playing critical roles in maintaining genomic stability and mediating chemotherapy resistance in tumor cells. The HRR mechanism utilizes undamaged sister chromatid DNA as a repair template, enabling high accuracy and efficiency in the repair process. This repair pathway is mediated by the serine/threonine kinase ATM and ATR, which activates Checkpoint Kinase 2 (CHK2) and subsequently regulates downstream effector molecules such as BRCA1, facilitating high‐fidelity DDR processes [23]. Research indicates that DNA DSBs pose serious threats to the integrity of cellular genetic material, where improper repair or repair failure may lead to cellular carcinogenesis or death. Defective DDR, driven by endogenous or exogenous factors, predisposes cells to replication stress and represents a critical determinant of cancer cell therapeutic vulnerability. In tumor cells, the assembly of the HR complex facilitates homologous recombination (HR); by modulating kinases to coordinate the DDR and suppress cell cycle progression, this mechanism counteracts genotoxic stress and repairs DSBs, thereby preserving genomic stability [24]. In the context of the cellular response to replication stress, ATR kinase plays a central role in activating DNA damage tolerance pathways. Impediments to replication fork progression—induced by replication stress or double‐strand breaks—result in helicase‐polymerase uncoupling and the subsequent generation of ssDNA at stalled forks. Replication protein A (RPA) coats the ssDNA at stalled forks and recruits ATR via its binding partner, ATR‐interacting protein (ATRIP). Upon activation by DNA topoisomerase II binding protein 1 (TopBP1), ATR phosphorylates CHK1 and suppresses cyclin‐dependent kinase 2 (CDK2) activity, thereby triggering ATR‐CHK1 pathway‐mediated S‐G2 cell cycle arrest and facilitating the HR process [25]. For instance, in oxaliplatin‐based therapy, widespread activation of the HRR mechanism repairs drug‐induced DNA damage, promoting the development of resistant phenotypes and significantly reducing chemotherapy efficacy. With the discovery of multiple targetable proteins, including ataxia telangiectasia mutated protein and CHK1/2, HRR and its related therapeutic strategies have emerged as important areas in precision oncology research. Homologous recombination deficiency (HRD) has been established as a predictive biomarker for PARPi therapy in ovarian cancer; furthermore, distinct HRD phenotypes correlate with somatic mutations and the epigenetic silencing of BRCA1 and BRCA2 in breast, prostate, and pancreatic cancers [26]. Furthermore, retrospective studies have demonstrated that HRR gene alterations hold significant value in molecularly guided therapy, with potential correlations between HRR gene mutation status (HRRm) and the efficacy of targeted therapies, offering critical guidance for personalized treatment of solid tumors [27].

### 2.2 NHEJ

NHEJ is an error‐prone repair mechanism initiated when DNA‐dependent protein kinase (DNA‐PK) complexes recognize and bind to DNA DSBs ends. The NHEJ repair process does not depend on homologous template DNA and occurs throughout all phases of the cell cycle. It repairs damage by directly joining broken DNA double‐strand ends; however, this repair method may lead to DNA sequence rearrangements, resulting in lower repair fidelity [28]. In HRD tumors, DNA DSBs are primarily repaired through the error‐prone NHEJ pathway, potentially counteracting the effects induced by chemotherapeutic agents. Key components of the NHEJ repair pathway include the Ku heterodimer (comprising Ku70 and Ku80) and the DNA‐PK catalytic subunit (DNA‐PKcs), which play a pivotal role in recognizing DSBs and activating downstream NHEJ signaling. During the initiation of NHEJ, the Ku heterodimer detects DNA break sites and binds to the broken ends; this binding induces a conformational change that recruits the DNA‐PK catalytic subunit, thereby assembling an active DNA‐PK complex [26]. Upon assembly, this complex undergoes autophosphorylation and mediates the phosphorylation of the XRCC4‐Ligase IV complex, XRCC4‐like factor (XLF), and XLF homologs. These events recruit core NHEJ proteins to the damage site to execute ligation, stabilize broken ends against nuclease degradation, and fully activate the NHEJ repair machinery [29]. While this process is crucial for maintaining genomic stability, it also mitigates the cytotoxic effects of DNA‐damaging agents—such as ionizing radiation (IR), IR mimetics, and certain alkylating agents—thereby potentially driving tumor progression and conferring cellular drug resistance. Specifically, studies indicate that in non‐melanoma skin cancer, the upregulation of Ku70 and Ku80 levels correlates with drug resistance, contributing to a poor prognosis. However, due to the low fidelity of the NHEJ repair pathway, genomic instability gradually accumulates throughout successive DNA replication cycles, eventually leading to NHEJ repair mechanism failure and triggering tumor cell death.

### 2.3 BER

BER is a crucial DNA repair mechanism primarily responsible for repairing SSBs caused by modified bases and oxidative damage, as well as minor lesions that do not significantly alter DNA structure. During the BER process, DNA glycosylases first recognize and excise damaged bases, AP endonuclease 1 (APE1) hydrolyzes the phosphodiester backbone at the AP site, generating a SSB characterized by a 3'‐hydroxyl group and a 5'‐deoxyribose phosphate (5'‐dRP) terminus. Subsequently, poly (ADP‐ribose) polymerase 1 (PARP1) recognizes the strand break, facilitating DNA end protection via poly‐ADP‐ribosylation (PARylation) and recruiting downstream effector proteins [30]. Mediated by DNA ligase I or the complexes of DNA ligase III and X‐ray repair cross‐complementing protein 1 (XRCC1), repair synthesis fills the gap, culminating in the covalent ligation of the nascent nucleotides to the parental DNA strand by DNA ligases [31]. The BER pathway plays a critical role in maintaining cellular genomic stability, with its repair function significantly reducing gene mutation frequency, thereby effectively preventing tumor development. Notably, the BER pathway plays a key role in addressing alkylating agent‐induced DNA damage, particularly through PARP‐dependent BER pathways or O6‐methylguanine removal processes mediated by the DNA repair enzyme MGMT, effectively resolving alkylation damage [32]. Consequently, owing to the protective activity of MGMT in malignancies with high expression levels—such as brain, skin, thymus, and liver cancers—significant resistance to exogenous alkylating agents is frequently observed [33].

### 2.4 NER

The NER system is primarily responsible for repairing bulky DNA adducts such as ICLs caused by alkylating chemotherapeutic agents and environmental carcinogens. The NER process comprises two distinct subpathways: global genome NER (GG‐NER) and transcription‐coupled NER (TC‐NER). GG‐NER initiates damage recognition via a complex consisting of Xeroderma Pigmentosum Complementation Group C (XPC), HR23B, and Centrin2; upon binding to chemically modified nucleotides, this complex recruits the XPF‐ERCC1 endonuclease complex to generate an exposed single‐stranded DNA (ssDNA gap within the double helix. Assisted by PCNA and replication factors, DNA polymerases perform DNA synthesis to fill the ssDNA gap, which is subsequently ligated by DNA ligase. Conversely, the TC‐NER pathway is initiated at sites of bulky DNA lesions, where Cockayne Syndrome Protein B (CSB) binds to stalled RNA polymerase and recruits Cockayne Syndrome Protein A (CSA) to induce chromatin remodeling, thereby activating the repair process [34]. The NER system is capable of repairing DNA damage induced by platinum‐based agents, a characteristic closely associated with the development of chemotherapy resistance in tumor cells. Mutations in the NER‐related gene ERCC2 may compromise cellular repair capacity, potentially promoting the progression of bladder cancer while attenuating cellular susceptibility to clinical therapeutic agents [35]. Currently, multiple therapeutic strategies targeting key NER enzymes have advanced to preclinical research stages and have demonstrated significant antitumor activity [36].

### 2.5 MMR

The MMR system is a highly conserved repair pathway comprising multiple genes and proteins that plays a crucial role in recognizing and repairing lesions such as base mismatches, nucleotide insertions, and deletions in DNA molecules. The MMR system utilizes MutS homologs to recognize mismatched nucleotides and activates MutH to cleave the unmethylated DNA strand, thereby transmitting the damage signal to downstream excision repair complexes. Exonucleases excise the nascent strand extending from the cleavage site to the mismatch; subsequently, DNA polymerase III and DNA ligase fill the resulting gap and seal the nick, thereby completing the repair process to maintain genomic integrity [37]. Deletion or inactivation of MMR‐related genes leads to ineffective repair of spontaneous mutations in repetitive DNA sequence regions, and this repair deficiency can alter the Tumor Microenvironment (TME) by activating oncogenes and suppressing tumor suppressor gene expression [38]. Notably, the functional status of the MMR signaling pathway is closely correlated with tumor susceptibility. Aberrant expression of key MMR genes significantly influences predisposition to colorectal and gastric malignancies, as well as the cellular proficiency in repairing chemotherapy‐induced DNA damage, ultimately leading to the development of chemotherapy resistance [27].

**Table:** 

DDR pathway	Key proteins/Genes involved	Mechanism of action in chemotherapy response	Targeted cancer types (examples)	Clinical/translational implications	References
**HRR** (Homologous Recombination Repair)	**BRCA1/2,** ATM, ATR	**Sensitivity**: Deficiency (HRD) prevents repair of platinum‐induced ICLs and DSBs. **Resistance**:Reversion mutations in BRCA1/2 or upregulation of RAD51 restores HRR function.	Ovarian Cancer, Breast Cancer, Prostate Cancer	Predictive biomarker for Platinum sensitivity and PARP inhibitors efficacy (e.g., Olaparib); Guidance for the personalized treatment of solid tumors	[24, 26]
**NHEJ** (Non‐ Homologous End Joining)	**DNA‐PKcs**, Ku70/80	**Sensitivity**: The NHEJ pathway is characterized by its low fidelity, which predisposes cells to genomic instability **Resistance**: Upregulation of DNA‐PKcs/Ku promotes rapid repair of DSBs induced by alky lating agents and IR	Non‐Melanoma Skin Cancer, Glioblastoma	High Ku70/80 levels correlate with poor prognosis; DNA‐PK inhibitors sensitize tumors to chemotherapy/radiotherapy.	[26, 29]
**BER** (Base Excision Repair)	**PARP1**, XRCC1, MGMT	**Sensitivity**: PARP trapping blocks BER, leading to toxic DSBs in S‐phase. **Resistance**: High MGMT levels directly remove alkyl groups (e.g., from temozolomide).	Malignancies of the brain, skin, thymus, and liver	MGMT promoter methylation status predicts Temozolomide response; PARP inhibitors target BER dependency.	[30, 31]
**NER** (Nucleotide Excision Repair)	**ERCC1**, ERCC2 (XPD), XPC	**Sensitivity**: Targeting key enzymes within the NER pathway to impede the DNA repair machinery **Resistance**: Enhanced removal of bulky platinum‐DNA adducts by ERCC1‐XPF complex reduces drug cytotoxicity.	Bladder Cancer	ERCC2 mutations may be associated with cellular repair capacity, thereby driving the progression of bladder cancer and reducing cellular susceptibility	[34, 35]
**MMR** (Mismatch Repair)	**MutS,** DNA polymerase III, DNA ligase	**Sensitivity**: dMMR causes high TMB, increasing neoantigens. **Resistance**: MMR deficiency leads to tolerance of alkylating agent‐induced mispairs	Colorectal and gastric malignancies	The deletion or inactivation of MMR‐related genes has the potential to modulate the tumor microenvironment	[37, 38]

## 3 Mechanisms Linking DDR to Tumor Chemosensitivity

**FIGURE 1:**
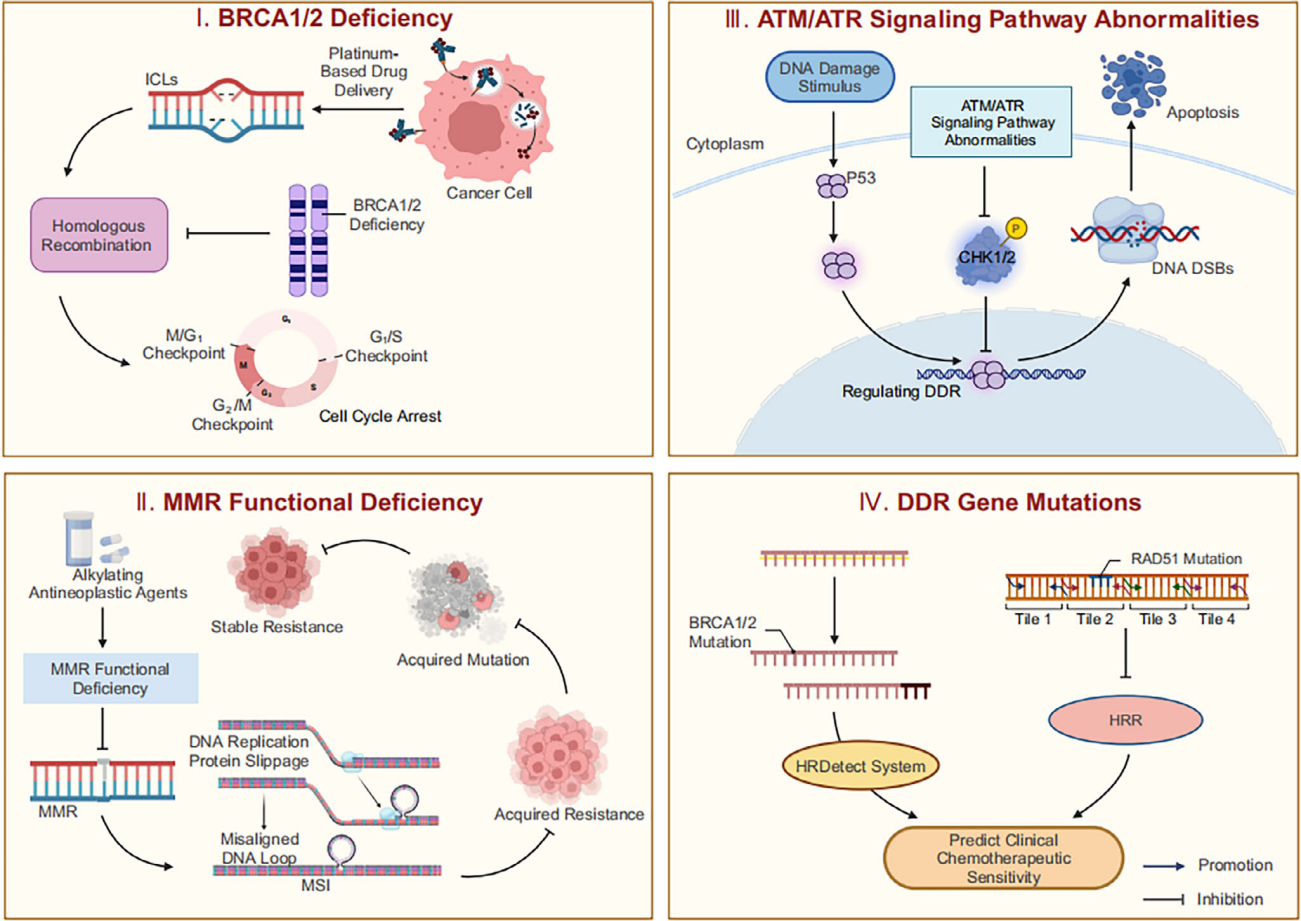
Mechanisms Linking DDR to Tumor Chemosensitivity. This figure was created using tools provided by Biorender.com (accessed on March 24, 2025). I. Platinum‐based therapies induce DNA inter‐strand cross‐links (ICLs), which exacerbate genomic instability and impede DNA replication. In BRCA1/2‐deficient tumor cells, the repair of these DNA cross‐links—which relies strictly on the BRCA1/2‐mediated homologous recombination repair (HRR) pathway—is compromised, ultimately triggering cell cycle arrest. II. Alkylating antitumor agents induce DNA alkylation modifications, interfering with DNA repair processes and triggering base mismatches. MMR functional deficiencies lead to ineffective DNA error correction, subsequently inducing MSI and significantly increasing single‐nucleotide substitution frequency, thereby suppressing the emergence of chemotherapy resistance. III. Dysfunction of the ATM/ATR signaling pathways impairs the activation of both ATM‐mediated homologous recombination repair (HRR) and non‐homologous end joining (NHEJ); this results in the accumulation of DNA double‐strand breaks (DSBs) within tumor cells, ultimately potentiating chemotherapy cytotoxicity. IV. Mutations in DNA damage repair (DDR) genes serve as valuable molecular biomarkers in oncological research. For instance, the HRDetect system predicts therapeutic responses to platinum‐based drugs by detecting homologous recombination repair (HRR) deficiencies stemming from BRCA1/2 mutations; similarly, alterations in the RAD51 gene can be used to evaluate the chemosensitivity of liver cancer cells.

Dysregulation of key genes or signaling pathways within the DDR system can significantly influence tumor chemosensitivity through various mechanisms (Figure 1). Among these, BRCA1/2 gene defects lead to HRR dysfunction, rendering tumor cells highly sensitive to platinum‐based agents; MMR system deficiencies enhance tumor cell sensitivity to alkylating agents by inducing Microsatellite Instability (MSI) and neoantigen generation; and ATM/ATR signaling pathway dysregulation impairs DDR capabilities, thereby exacerbating genomic instability. Notably, DDR gene mutations not only serve as molecular markers for predicting chemotherapy response (such as the HRDetect prediction system) but may also stimulate anti‐tumor immune responses by increasing Tumor Mutational Burden (TMB). These findings provide a solid theoretical foundation for developing personalized chemotherapy strategies based on DDR status and for elucidating resistance mechanisms.

### 3.1 BRCA1/2 Deficiency

As critical genes in DNA DSBs repair, BRCA1/2 play central roles in regulating the HRR pathway, and their functional deficiencies are closely associated with the development and progression of multiple tumors. Research has demonstrated that tumors harboring BRCA1/2 mutations exhibit significant sensitivity to platinum‐based drugs, with these BRCA1/2‐deficient tumors typically demonstrating higher response rates to platinum‐based therapy and improved prognosis [6]. Platinum‐based drug treatment induces the formation of DNA ICLs, increases genomic instability, and subsequently triggers tumor cell apoptosis. The repair of such DNA ICLs damage is critically dependent on the BRCA1/2‐mediated HRR pathway [39]. BRCA1/2 functional loss leads to impaired HRR pathways [40], thereby preventing cells from effectively repairing platinum‐induced DNA damage, resulting in cell cycle arrest, and ultimately enhancing tumor cell sensitivity to platinum‐based chemotherapy. Therefore, BRCA1/2 gene mutation status functions as an important molecular marker for predicting sensitivity to platinum‐based therapy, providing a theoretical basis for developing personalized treatment strategies.

### 3.2 MMR Functional Deficiency

Alkylating antineoplastic agents lead to DNA SSBs and DSBs by inducing DNA alkylation modifications, which inhibit tumor cell proliferation and division. However, the MMR mechanism in tumor cells rapidly repairs damaged DNA, representing a major mechanism contributing to chemotherapy resistance. Research demonstrates that MMR functional deficiency significantly enhances tumor cell sensitivity to alkylating agents. MMR functional deficiency leads to an increased frequency of polymorphic short repeat DNA sequences and single nucleotide substitutions, thereby triggering MSI. Simultaneously, neoantigens produced by numerous high‐frequency non‐synonymous and frameshift mutations further enhance tumor cell sensitivity to alkylating agents by inhibiting single and double‐strand DNA repair processes and eliciting stable, sustained immune responses [41].

### 3.3 ATM/ATR Signaling Pathway Abnormalities

ATM and ATR are critical kinases in the DDR process, and disruptions in their signaling pathways are closely associated with tumor cell chemosensitivity. DNA DSBs simultaneously activate both ATM and ATR signaling pathways, with ATR primarily regulating DDR and cell cycle checkpoint activation, thereby inducing cell cycle arrest [42]. ATM responds to DNA DSBs by phosphorylating protein kinases such as CHK1, initiating cascading reactions of downstream effector molecules, thus providing tumor cells with sufficient time for DDR. When defects occur in the ATM/ATR signaling pathway, both ATM‐mediated HRR and NHEJ repair pathways fail to activate properly, resulting in decreased DSBs repair efficiency, accumulation of intracellular DNA damage, and ultimately increased tumor cell sensitivity to chemotherapeutic agents [43].

### 3.4 DDR Gene Mutations

DDR gene mutations, as molecular markers of significant interest in tumor biology research, possess substantial value in predicting clinical chemotherapeutic sensitivity [44]. Research shows that mutations in DNA repair genes alter tumor cell drug sensitivity by affecting DDR functionality. For instance, the HRDetect system, used to detect HRR deficiency caused by BRCA1/2 mutations, has been successfully applied to predict tumor response to platinum‐based agents; additionally, RAD51 gene alterations significantly reduce tumor cell migration and invasion, and increase hepatocellular carcinoma sensitivity to chemotherapeutic agents by blocking HRR [45]. Therefore, findings from DDR gene mutation research provide an essential theoretical basis for developing clinical chemotherapy regimens. Additionally, DDR gene mutations correlate with TMB, an association that offers potential for new therapeutic strategies to improve patient prognosis by stimulating innate anti‐tumor immune responses. Whole‐genome analysis studies of Non‐Small Cell Lung Cancer (NSCLC) demonstrate that DDR gene mutations are closely associated with the sensitivity of various solid tumors to chemotherapeutic agents, highlighting the clinical utility of DDR mutations as potential biomarkers [46]. As predictive biomarkers of treatment response, DDR gene mutations enable assessment of chemotherapy sensitivity through mutation status detection and, when combined with tumor heterogeneity analysis of treatment response differences, provide a critical basis for achieving precision personalized cancer therapy [47].

## 4 Mechanisms Linking DDR to Tumor Chemotherapy Resistance

**FIGURE 2:**
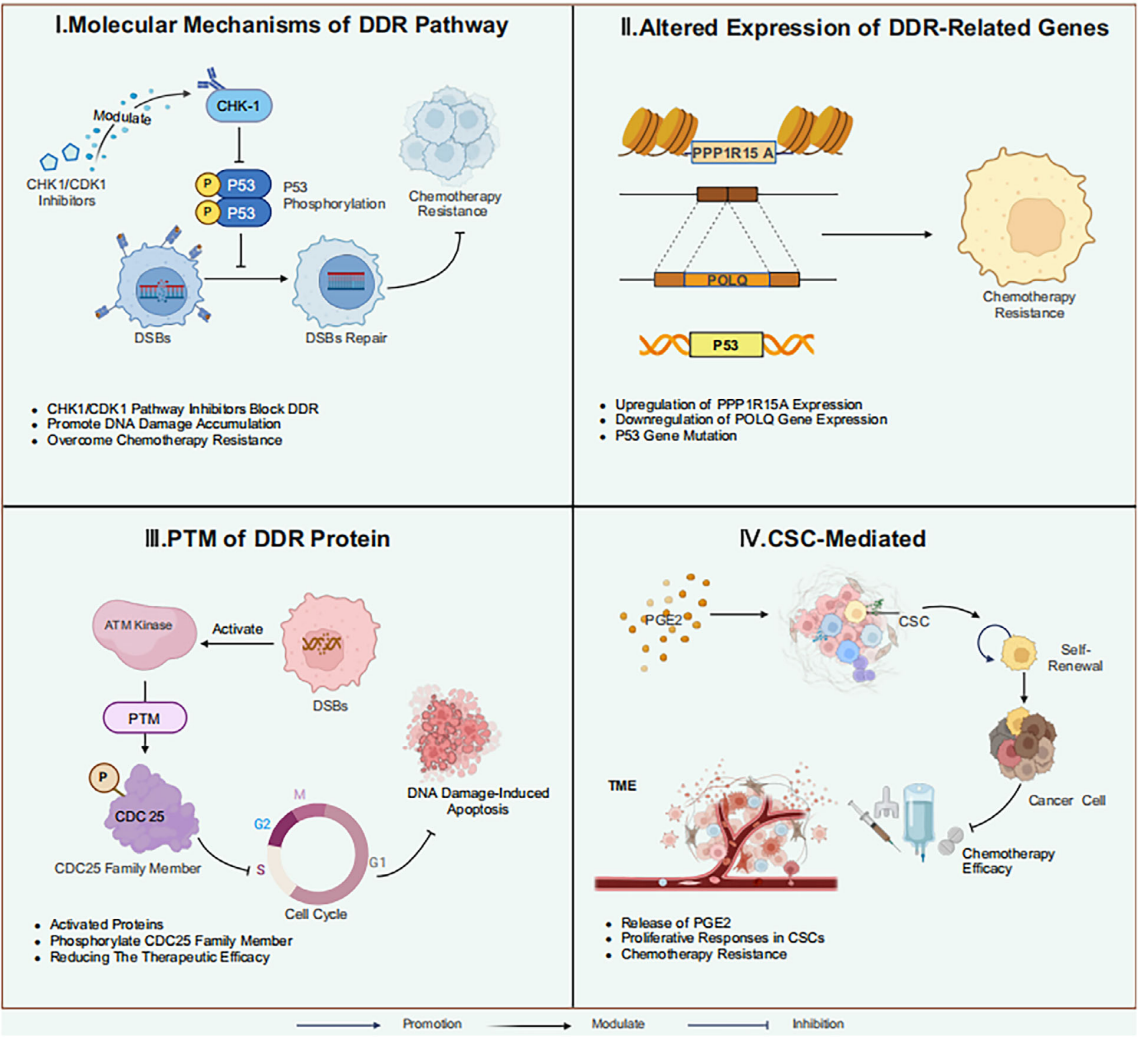
Mechanisms Linking DDR to Tumor Chemotherapy Resistance. This figure was created using tools provided by Biorender.com (accessed on March 24, 2025). I. CHK1/CDK1 inhibitors block the CHK1/CDK1 signaling pathway, thereby inhibiting p53 phosphorylation and preventing the activation of p53‐dependent DNA damage checkpoints. This inhibition impairs the DDR capacity of tumor cells, promotes intracellular DNA damage accumulation, and consequently overcomes chemotherapy resistance mediated by DDR pathway activation. II. Alterations in the expression levels of DDR‐related genes may enhance DDR capacity in tumor cells, leading to cellular resistance to chemotherapeutic agents. For instance, significant upregulation of PPP1R15A expression affects the activity of DNA repair pathways, downregulation of the POLQ gene can promote the activation of RAD51 protein‐mediated DNA repair pathways, and mutations in the p53 gene can cause cell cycle checkpoint failures—all of which can decrease tumor cell sensitivity to chemotherapeutic drugs and induce the development of resistance. III. When chemotherapeutic agents induce persistent DNA DSBs, activated proteins such as ATM and p53 directly phosphorylate CDC25 family members through PTM processes, thereby promoting cell cycle arrest in tumor cells. This arrest subsequently results in reduced therapeutic efficacy of chemotherapeutic agents against these malignant cells. IV. During DNA damage, dying tumor cells can release PGE2 through paracrine signaling pathways, thereby activating proliferative responses in CSCs. These stem cells not only intrinsically resist DNA damage‐induced apoptosis but can also develop acquired resistance phenotypes under the regulation of the TME, ultimately promoting tumor cell survival and exacerbating the development of chemotherapy resistance.

Research confirms that aberrant activation of DDR signaling pathways represents one of the key molecular mechanisms underlying tumor chemotherapy resistance (Figure 2). DNA damage induced by classical chemotherapeutic agents triggers cell cycle arrest through the ATM/ATR‐CHK1 signaling cascade, thereby providing a time window for DDR. Meanwhile, altered expression levels of DDR‐related genes (such as POLQ and p53) significantly enhance DNA repair capacity or disrupt cell cycle checkpoint function, ultimately leading to decreased tumor cell sensitivity to chemotherapeutic agents. More importantly, post‐translational modifications of DDR‐related proteins (including phosphorylation, ubiquitination, etc.) promote tumor cell resistance development by regulating the activity levels of repair proteins. Particularly noteworthy is that Cancer Stem Cells (CSCs) establish resistance barriers not only through their inherent anti‐apoptotic characteristics but also by activating proliferative responses through stimulation by microenvironmental signaling molecules (such as Prostaglandin E2[PGE2]). Based on these mechanisms, targeted inhibition strategies against key nodes in DDR pathways (such as CHK1/CDK1) and the CSCs microenvironment offer novel therapeutic approaches for overcoming tumor resistance.

### 4.1 Molecular Mechanisms of DDR Pathways in Chemotherapy Resistance

DDR mechanisms effectively repair chemotherapy‐induced DNA damage, thereby inhibiting cell apoptosis. This enhanced activity of DDR pathways is closely associated with the emergence of chemotherapy resistance. Research shows that activation of the CHK1/CDK1 signaling pathway leads to NSCLC cell cycle arrest, thereby enhancing DDR capacity, attenuating cisplatin (DDP) cytotoxicity, and ultimately resulting in tumor cell resistance [48]. From a molecular mechanism perspective, chemotherapy‐induced DNA DSBs activate the ATM/ATR signaling pathway, promoting CHK1 phosphorylation, subsequently activating p53‐dependent DNA damage checkpoints, inducing cell cycle arrest, and providing an adequate time window for DNA repair. Research has revealed that CHK1/CDK1 pathway inhibitors, such as the sponge‐derived alkaloid AP‐7, block DDR, promote DNA damage accumulation, and thereby overcome chemotherapy resistance mediated by DDR pathway activation.

### 4.2 Altered Expression of DDR‐Related Genes and Chemotherapy Resistance

Sustained normal expression of DDR‐related genes plays a central role in maintaining genomic stability, and alterations in their expression levels are closely associated with chemotherapy resistance in tumor cells. Altered expression of DDR‐related genes enhances the DDR capacity of tumor cells, thereby conferring resistance to chemotherapeutic agents. For instance, significant downregulation of PPP1R15A expression modulates the activity of DNA repair pathways, enhances AML cell sensitivity to idarubicin or cytarabine treatment, thereby inhibiting the development of resistance [49]. Similarly, downregulation of POLQ gene expression enhances the activity of RAD51 protein‐mediated DNA repair pathways, thereby increasing tumor cell resistance to platinum‐based agents [50]. Additionally, altered expression of DDR genes impairs cell cycle checkpoint function, allowing damaged tumor cells to continue proliferating. A typical example is that p53 gene mutation or functional loss leads to cell cycle checkpoint failure [51, 52], ultimately decreasing tumor cell sensitivity to chemotherapeutic agents and promoting resistance development.

### 4.3 Association Between Post‐Translational Modification (PTM) of DDR Proteins and Chemotherapy Resistance

The DDR system maintains genomic stability by orchestrating complex protein networks in response to DNA damage events. However, the modification mechanisms of these proteins reduce the therapeutic efficacy of chemotherapeutic agents, thereby promoting tumor cell resistance. PTM, as a key regulatory mechanism after protein synthesis, is widely involved in various cellular biological processes, plays a crucial role in the relationship between DDR and chemotherapy, and constitutes one of the core mechanisms regulating protein function [53]. The PTM process of DDR‐related proteins primarily regulates protein activity and function through covalent bonding of chemical groups, amino acids, or other complex molecules, a process intricately associated with tumorigenesis and chemotherapy resistance. Phosphorylation is the most prevalent and important PTM, with significant implications for the relationship between DDR and chemotherapy efficacy. When cells experience persistent DSBs or SSBs induced by chemotherapeutic agents, proteins such as ATM and p53 are activated, and these activated proteins directly phosphorylate CDC25 family members, inducing cell cycle arrest in tumor cells while influencing timely repair of DNA damage, ultimately reducing the therapeutic efficacy of chemotherapeutic agents on tumor cells and promoting cellular resistance [48]. Furthermore, other PTM processes such as ubiquitination and methylation modulate DDR by modifying key proteins, which is significant for a deeper understanding of the emergence of resistance during chemotherapy [54].

### 4.4 DDR Pathways and CSC‐Mediated Chemotherapy Resistance Mechanisms

CSCs are a distinct subpopulation of tumor cells with self‐renewal capacity and the potential to reconstitute the heterogeneity of primary tumors. The development of tumor chemotherapy resistance is primarily attributed to the presence of CSCs, which not only possess intrinsic resistance to DNA damage‐induced apoptosis but also acquire adaptive resistance characteristics under the regulation of the TME. Although CSCs with metastatic potential may exhibit phenotypic differences from tumor cells that have acquired resistance, the inherent chemoresistance characteristics of CSCs represent key factors leading to tumor recurrence. Clinical studies demonstrate that DNA damage triggers the release of PGE2 from dying tumor cells after chemotherapy through paracrine signaling pathways, thereby activating proliferative responses in CSCs [55], ultimately promoting tumor cell survival and disease progression. Additionally, the TME—comprising hematopoietic‐derived cells, mesenchymal cells, and acellular components—interacts with CSCs through diverse signaling pathways and molecular mechanisms, fostering therapeutic resistance in CSCs and compromising chemotherapy efficacy [56]. Studies indicate that the modulation of hypoxia, dysregulated angiogenesis, and distinct niches constitutes a critical interaction mode between the TME and CSCs; these mechanisms confer cellular plasticity and heterogeneity, driving treatment failure. Crosstalk between CSCs and immune cells is mediated by immune checkpoints and exosomes. CSC‐derived exosomes transport bioactive cargo, including specific proteins and transcription factors, delivering functional molecules to immune cells to exert potent immunomodulatory effects. Exosome‐mediated niches generated by CSCs can indirectly regulate pre‐metastatic niche formation, contributing to the maintenance of stemness and facilitating immune evasion [57]. In one study, CSCs promoted the polarization of CD14+ peripheral monocyte‐derived macrophages toward an immunosuppressive phenotype within a co‐transplantation setting, resulting in accelerated tumor growth in immunocompromised mice [58]. During chemotherapy, CSCs under stress conditions establish microenvironments favorable for cell survival through proliferative response mechanisms while impeding contact between drugs such as gemcitabine and tumor cells, further exacerbating the development of chemotherapy resistance. Therefore, elucidating CSCs resistance mechanisms and their regulatory networks is crucial for developing therapeutic strategies targeting CSCs and overcoming resistance that emerges during tumor chemotherapy [59].

## 5 Chemosensitization Strategies Targeting DDR

DDRi, by selectively targeting defects in DDR pathways, can enhance the sensitivity of tumor cells to chemotherapeutic agents, thereby overcoming chemoresistance, and have consequently emerged as a highly promising therapeutic strategy for cancer treatment [34]. Elucidating the molecular regulatory mechanisms that link DNA repair pathways to tumor resistance is of significant importance for developing novel therapeutic strategies [60, 61, 62].

### 5.1 PARPi

The PARP protein superfamily, functioning as key DDR factors, exerts multiple regulatory functions in cellular biological processes. Current research indicates that the eight identified members of the PARP superfamily are deeply involved in the repair processes of DNA SSBs and DSBs, as well as cell cycle regulation [63]. These proteins effectively repair DNA damage caused by chemotherapeutic agents (especially alkylating agents), a mechanism that directly leads to chemoresistance in tumor cells. Advances in the in‐depth elucidation of the molecular mechanisms of HRD have significantly accelerated the development of PARPi [34]. Research demonstrates that PARPi, by specifically blocking DDR pathways, significantly enhance the sensitivity of tumor cells to chemotherapeutic agents. As targeted therapeutic agents that promote DNA damage accumulation by interfering with DNA repair mechanisms, PARPi achieve their antitumor effects primarily by inhibiting the catalytic activity of PARP enzymes, inducing PARP1/2 protein trapping, and blocking SSBs repair mediated by BER [23]. Their mechanism of action primarily involves blocking PARP‐mediated poly (ADP‐ribosyl)ation (PARylation) of both PARP proteins themselves and other DDR substrates, thereby effectively interfering with DDR and significantly affecting cell proliferation and survival [64]. Based on differences in their mechanisms of action, PARPi can be classified into two categories: (1) potent inhibitors such as olaparib that primarily “trap” PARP proteins on chromatin, leading to cytotoxic damage and impaired repair of DNA SSBs, ultimately causing DNA damage accumulation and replication fork collapse, thereby triggering PARP/BRCA synthetic lethality. The PARP trapping theory postulates that PARP inhibitors competitively bind to the NAD+‐binding domain of PARP1, thereby inhibiting its catalytic activity. Consequently, prevented from undergoing auto‐PARylation, PARP1 becomes entrapped on DNA, causing stalled replication forks and DSBs at damage sites as cells progress through the S phase. In HR‐deficient tumor cells, where DSB repair relies on compensatory NHEJ, genomic instability is exacerbated, driving the synthetic lethality associated with BRCA1 deficiency and PARP inhibition [65]. (2) conversely, allosteric inhibitors, exemplified by veliparib, induce conformational changes in PARP1 that attenuate DNA binding and promote its dissociation from DNA [66]. Furthermore, through structure‐guided optimization, allosteric inhibitors can be engineered to shift from promoting release to enhancing retention, thereby conferring potent PARP1 trapping potency and increased cytotoxicity against cancer cells [67]. Based on the high dependency of BRCA1/2‐mutated tumors on PARP proteins, PARPi have demonstrated significant clinical efficacy in treating HRD tumors, effectively overcoming chemoresistance and significantly prolonging patient survival [68]. Therefore, PARPi have emerged as a novel targeted therapeutic strategy with significant clinical application value in contemporary cancer treatment.

### 5.2 ATM/ATR Inhibitors

DDR is a cellular defense mechanism that is precisely regulated by a complex network of signal transduction proteins. Among these, ATM and ATR kinases function as core regulatory factors of the DDR signaling pathway. These kinases induce cell cycle checkpoint arrest and regulate downstream effector molecules, enhancing DSBs repair capacity, ultimately leading to acquired resistance of tumor cells to DNA‐damaging anticancer drugs [69]. To overcome this clinical challenge, selective small molecule inhibitors targeting ATM/ATR have become a promising avenue in antitumor drug development and have demonstrated significant clinical potential. ATM/ATR inhibitors specifically target key kinases in the DDR pathway, thereby interfering with the cell cycle checkpoint regulatory network in tumor cells. Among these, the natural compound Shikonin specifically induces proteasome‐dependent degradation of ATM‐ATR Interacting Protein (ATRIP), inhibiting the activation of upstream regulatory factors in the DDR signaling pathway, thereby enhancing the sensitivity of tumor cells to chemotherapeutic agents [70]. Research indicates that the combined application of ATR inhibitors and DNA damage inducers triggers synthetic lethal effects, significantly enhancing the antitumor activity of chemotherapeutic agents such as cisplatin and the PARPi veliparib [71], the highly selective ATR inhibitor Berzosertib has demonstrated efficacy in suppressing pancreatic tumor progression while exhibiting minimal off‐target toxicity in normal tissues. Preliminary data from Phase I trials involving advanced solid tumors suggest that combining Berzosertib with the platinum‐based agent Cisplatin exhibits a favorable safety and tolerability profile [72]. Current clinical trials are evaluating the safety, pharmacokinetics, and preliminary antitumor efficacy of the potent ATM inhibitor WSD0628 in treating persistent brain tumors [73], thus providing new therapeutic approaches for optimizing clinical combination drug strategies [74].

### 5.3 CHK1/2 Inhibitors

CHK1/2, serving as key downstream effector molecules of the ATR and ATM signaling pathways, primarily trigger cell cycle arrest by precisely regulating G1/S and G2/M checkpoints, thereby exerting core regulatory functions in the DDR network of tumor cells [75]. Recent studies have confirmed that small molecule inhibitors specifically targeting CHK1/2 both effectively reverse chemoresistance in tumor cells and significantly enhance their sensitivity to chemotherapeutic agents. In solid tumor treatment, CHK1 inhibitors primarily interfere with the assembly of multiprotein complexes at single‐strand DNA damage sites, thereby blocking the activation of the ATR‐CHK1 signaling axis, ultimately inducing synthetic lethal effects [43]. Additionally, CHK2 inhibitors specifically interfere with DDR pathways in tumor cells and promote tumor cell apoptosis by disrupting cell cycle regulatory networks, effectively inhibiting tumor progression, thereby significantly enhancing the therapeutic effects of conventional chemotherapeutic agents. Notably, by selectively inhibiting checkpoint kinase activity, CHK2 inhibitors significantly enhance the sensitivity of tumor cells to DNA‐damaging drugs, effectively overcome chemoresistance, and provide a highly promising therapeutic strategy for improving patient prognosis [76]. Preclinical studies have investigated the efficacy of the CHK1/2 inhibitor prexasertib combined with cisplatin against head and neck squamous cell carcinoma. Results indicated that prexasertib abrogated chemotherapy‐induced checkpoint activation and modulated cell cycle distribution, resulting in sustained DNA damage; these findings highlight the potential for enhanced clinical benefits and offer a rationale for developing novel therapeutic strategies [77].

### 5.4 DNA‐PK Inhibitors

DNA‐PKcs not only serves as a key enzyme in the NHEJ repair pathway but also mediates the repair of chemotherapy‐induced DNA DSBs by forming complexes with DNA‐binding proteins Ku70/80, ultimately leading to resistance in tumor cells [78]. Recent studies have shown that DNA‐PK inhibitors specifically block the binding of DNA‐PKcs to DNA damage sites, thereby enhancing the cytotoxic effects of chemotherapeutic agents. In in vivo studies, quantitative detection of changes in EdU‐positive cells in mouse jejunum tissue before and after drug administration has confirmed that DNA‐PK inhibitors significantly reduce the tolerance of tumor cells to etoposide. These research findings indicate that the combined use of DNA‐PK inhibitors with chemotherapeutic agents produces significant chemosensitizing effects [79]. A preliminary clinical trial demonstrated the therapeutic efficacy of the DNA‐PK inhibitor CC‐115 in patients with brain tumors and diverse malignancies, positioning it as a promising candidate for future anticancer interventions [80]. However, owing to modest efficacy or significant toxicity, certain inhibitors, such as XL765, have exhibited limited utility in clinical trials and remain unsuitable for clinical application [81].

### 5.5 Other DDRi

As research on the regulatory mechanisms of DDR networks deepens, various small molecule inhibitors targeting DDR signaling pathways have entered active development stages. Research indicates that Lactate Dehydrogenase (LDH) inhibitors enhance tumor treatment efficacy by blocking the conversion of pyruvate to lactate, thereby inhibiting the expression of NER‐related genes [82]. Protein arginine methyltransferase 5 (PRMT5) inhibitors significantly downregulate the expression levels of various DDR‐related genes in cancer cells, thereby increasing the sensitivity of cancer cells to chemotherapeutic agents [83]. WEE1 kinase inhibitors induce cell apoptosis by promoting the entry of cells carrying unrepaired DNA damage into mitosis. Therefore, optimizing existing treatment regimens and developing combination therapy strategies with novel DDRi offers promising potential to significantly enhance the clinical therapeutic efficacy of chemotherapy [84].

## 6 Future Perspectives

**FIGURE 3:**
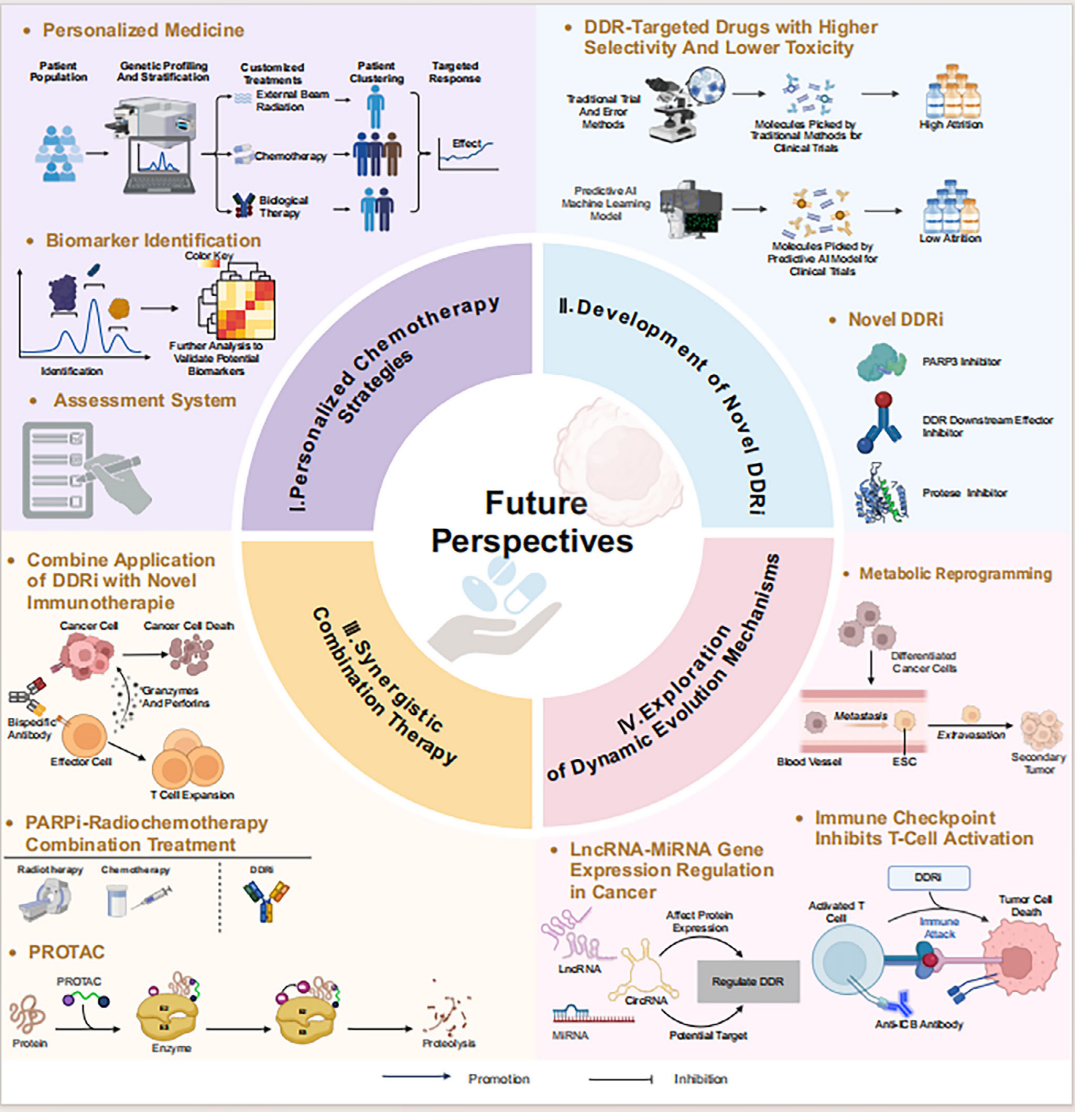
Future Perspectives of DDR research. This figure was created using tools provided by Biorender.com (accessed on March 24, 2025). I. Due to the variation in treatment responses arising from diverse molecular pathological characteristics, chemotherapy personalization strategies based on precise DDR classification are increasingly emerging as research hotspots in tumor treatment. Identifying and validating molecular markers for predicting drug sensitivity, establishing functional evaluation systems for DDR pathways, and developing highly sensitive DDR deficiency detection technologies will significantly enhance treatment precision. II. Selecting DDR‐targeted drugs with enhanced selectivity and reduced toxicity in clinical settings holds promise for providing novel therapeutic interventions for tumor treatment. For example, novel inhibitors targeting DNA damage sensing proteins, such as PARP3, can induce DNA damage accumulation, while small‐molecule inhibitors targeting DDR downstream effectors demonstrate potential to reverse chemotherapy resistance. III. In‐depth exploration of synergistic mechanisms between DDR pathways and novel therapies offers significant clinical value. For instance, the combination of DDRi with novel immunotherapies, such as bispecific antibodies, shows promising application value; the safety and feasibility of PARPi‐radiochemotherapy triple therapy have been validated; and combining DDRi with PROTAC technology can effectively improve patient outcomes. IV. Exploring the dynamic evolution mechanisms of DDR‐mediated chemotherapy resistance, systematically analyzing the relationship between DDR pathways and metabolic reprogramming, investigating the regulatory networks between DDR and non‐coding RNAs, and developing DDR‐regulated ICB therapies not only have important implications for enhancing clinical treatment outcomes, but also represent potential strategies for reversing resistance and improving chemosensitivity.

Although research into DDR is ushering in a new era of precision medicine, current progress remains constrained by several limitations: the molecular heterogeneity of tumor DDR remains to be fully elucidated, the toxicity profiles of DDR inhibitors have not been completely resolved, the safety of synergistic treatment paradigms requires rigorous exploration, and the interplay between DDR gene mutations and the tumor immune microenvironment remains ill‐defined. To address these challenges, future research and clinical translation should prioritize the implementation of personalized chemotherapy strategies through systematic DDR pathway profiling, the continued development of novel DDR inhibitors to reverse chemotherapy resistance, the comprehensive investigation of synergistic paradigms involving DDR, immunotherapy, and radiotherapy, and the systematic elucidation of the dynamic evolutionary mechanisms by which DDR modulates chemotherapy resistance. These research advances provide multidimensional innovative directions for enhancing the precision and effectiveness of cancer treatment, with the potential to transform traditional cancer chemotherapy models and fundamentally advance precision cancer therapy into a new era (Figure 3).

### 6.1 Personalized Chemotherapy Strategies Based on Precise DDR Classification

In the context of rapidly developing precision medicine, different treatment responses triggered by varying molecular pathological characteristics have made individualized therapeutic strategies targeting DDR pathways an increasingly important direction in cancer treatment [85]. In this field, identifying and validating molecular biomarkers that predict drug sensitivity in cancer patients is of significant importance for enhancing therapeutic efficacy and reducing drug toxicity [86]. However, due to the complexity of signaling pathway networks and the existence of tumor molecular heterogeneity, accurately determining the molecular pathogenic mechanisms in individual patients still faces significant challenges [87]. For instance, the predictive utility of HRD, a currently widely utilized biomarker, may be compromised by resistance mechanisms arising from prior treatments. To address these challenges, researchers are actively exploring innovative therapeutic strategies and technical platforms to elucidate the association between DDR gene polymorphisms and therapeutic responses. Clinical trials are continuously evaluating key predictive factors—such as genomic scar scores, BRCA1/2 promoter methylation, and RAD51 loading—while analyzing critical data regarding somatic status, allele‐specific copy numbers, and germline characteristics, thereby achieving dynamic monitoring of HRD function. This approach may profoundly refine biomarker correlations and facilitate the construction of a multi‐dimensional biomarker‐based assessment system for DDR pathway function, ultimately maximizing the efficacy of individualized treatment strategies. Given the limitations imposed by primary and acquired drug resistance, the therapeutic efficacy and durability of clinical drug combinations often fail to realize truly personalized treatment strategies; therefore, the development of technologies, such as the high‐sensitivity detection of DDR defects, is required to precisely screen combination regimens tailored to individual patient profiles, thereby enhancing treatment precision [88, 89]. CRISPR functional genomic screening technology, based on the concept of synthetic lethality, enables the comprehensive functional genomic assessment of all known cancer genes, facilitating the systematic screening of synthetic lethal drug targets in human cancer. Through anchor screens, the CRISPR system can be applied to identify drug combinations involving novel targets, providing a promising pathway for establishing the biomarker context for patient selection in clinical trial development [90].

### 6.2 Development of Novel DDRi to Overcome Chemoresistance

As understanding of DDR pathways deepens, developing inhibitors targeting novel DDR targets provides new research directions for breaking through existing therapeutic bottlenecks. In the field of DNA damage sensor protein research, although PARP1 has received considerable attention, recent studies have found that PARP3 expression levels significantly correlate with cancer patient prognosis in the TCGA database, and targeting PARP3 induces DNA damage accumulation and modulates immune responses [91]. Therefore, developing novel inhibitor strategies targeting DNA damage sensor proteins such as PARP3 holds promise for providing new intervention approaches for clinical treatment. Additionally, although the development of small molecule inhibitors targeting DDR downstream effectors is relatively lagging, these compounds have significant potential in reversing chemoresistance and improving treatment efficacy. However, existing DDRi generally suffer from issues such as insufficient selectivity and systemic toxicity, commonly causing adverse events such as diarrhea, fatigue, nausea, neutropenia, and leukopenia. Mechanisms including the restoration of BRCA1/2 function, stabilization of replication forks, and drug target‐related effects frequently drive acquired resistance, limiting their clinical application. Therefore, developing next‐generation DDR‐targeted drugs with higher selectivity and lower toxicity is of significant importance for enhancing clinical therapeutic outcomes [92]. To mitigate chemotherapy‐associated toxicity and develop DDR strategies capable of overcoming resistance, a key approach involves the selective delivery of chemotherapeutic agents. For instance, CRLX101 uses a nanoparticle formulation for preferential delivery to tumors via leaky vasculature, demonstrating an improved safety profile compared to conventional Top1 inhibitors such as topotecan and irinotecan. Continued investigation into the antitumor efficacy and tolerability of combining chemotherapeutic agents with DDR inhibitors is of significant clinical importance [11]. Other potential approaches to address therapeutic challenges associated with novel DDR inhibitors include implementing combination therapies to potentiate antitumor effects and inhibiting mutator phenotypes to delay resistance onset.

### 6.3 Paradigms of Synergistic Combination Therapy with DDR and Chemotherapy

Research on the synergistic effects between DDR pathways and novel treatment modalities provides an important theoretical foundation for developing innovative combination therapy regimens. The combined application of DDRi with traditional immunotherapy has shown significant therapeutic efficacy; however, the synergistic mechanisms underlying their interactions with novel immunotherapies still require in‐depth exploration. Furthermore, although the synergistic mechanisms between novel immunotherapeutic approaches such as bispecific antibodies and DDRi have potential application value, their molecular mechanisms remain to be elucidated [85]. Given the close relationship between radiotherapy efficacy and DNA DSBs repair mechanisms, DDRi can improve patient prognosis by enhancing radiosensitivity. Radiation‐induced targeted DNA damage provides a theoretical basis for triple therapy combining DDR regulation with targeted therapy and radiotherapy [93]. It should be noted that although PARPi‐radiochemotherapy combination treatment has demonstrated safety and feasibility, the patient population that benefits is limited. Therefore, exploring combinations of DDRi with emerging therapeutic approaches such as Proteolysis Targeting Chimera (PROTAC) technology has significant clinical value [94]. By facilitating the degradation of target proteins, DDR‐targeting PROTACs can circumvent resistance mechanisms arising from mutations or alternative signaling pathways, thereby imparting enhanced selectivity to traditional DDR inhibitors. To promote the clinical translation of PROTAC technology, it is imperative to thoroughly elucidate the biological functions and regulatory mechanisms of potential targets, evaluate the off‐target effects and toxicities associated with targeting specific DDR proteins, and maximize the clinical potential of these emerging therapeutic modalities [95].

### 6.4 Elucidating the Dynamic Evolution of DDR‐Mediated Drug Resistance

DDR plays complex and critical roles in tumor progression and metastasis by regulating DDR processes, promoting tumor cell recognition and repair of chemotherapy‐induced DNA damage, enhancing adaptation to genotoxic stress, and maintaining genome stability in CSCs. As an emerging mechanism, metabolic reprogramming transforms differentiated cells into states with Embryonic Stem Cell (ESC) characteristics. Autophagy is closely associated with tumor metastasis and recurrence, although its exact role remains controversial [96]. Meanwhile, understanding of chemosensitivity and resistance mechanisms and their associated biomarkers still requires further investigation. In‐depth exploration of the relationship between DDR pathways and CSCs self‐renewal and differentiation [97], as well as developing novel anti‐metastatic strategies targeting DDR, is particularly important [98]. Due to the complex regulatory networks between DDR and non‐coding RNAs, precision therapeutic strategies targeting Long non‐coding RNAs (LncRNAs) have become a frontier area in cancer treatment research. Current research shows that MicroRNAs (MiRNAs) regulate DDR processes by precisely controlling the expression and activation of DDR‐related proteins [99], while the loss of Circular RNA (CircRNA) expression or disruption of CircRNA‐MRNA interactions inhibits DDR processes [100]. These non‐coding RNAs are thus emerging as potential targets for optimizing chemotherapy efficacy. Furthermore, with the deepening clinical application of immunotherapy, numerous studies have confirmed that DNA repair mechanisms significantly affect the sensitivity and therapeutic response to Immune Checkpoint Blockade (ICB) therapy [101], revealing complex interaction networks between DNA repair and immune cell infiltration. Genetic mutations in DDR are closely related to the remodeling of the tumor immune microenvironment, as evidenced by higher tumor mutation burden frequently observed in MMR deficiency [102]. Although the clinical benefits of ICB therapy exhibit significant heterogeneity and limitations, developing strategies based on DDR regulation to reshape the tumor immune microenvironment advances cancer treatment processes [103], offering substantial clinical value. Numerous ongoing clinical trials are evaluating the safety and efficacy of combining DNA repair agents with immune checkpoint blockade across diverse dosing and scheduling regimens, seeking to address the context‐dependent variability in optimal timing.

## Author Contributions

Anqi Lin, Jinyue He, and Aimin Jiang are the joint authors. Anqi Lin, Jinyue He, and Aimin Jiang contributed equally to this work and share first authorship. Anqi Lin, Jinyue He, Jian Zhang, Quan Cheng, Hengguo Zhang, Wenjun Mao, and Peng Luo: writing – original draft. Hengguo Zhang, Wenjun Mao, and Peng Luo: conceptualization and investigation. Anqi Lin, Jinyue He, Aimin Jiang, Jian Zhang, Quan Cheng, Hengguo Zhang, Wenjun Mao, and Peng Luo: writing – review and editing. Anqi Lin, Jinyue He, and Aimin Jiang: visualization. All authors have read and agreed to the published version of the manuscript.

## Conflicts of Interest

The authors declare no conflicts of interest.

## Peer Review

The peer review history for this article is available in the Supporting Information for this article.

## Supporting information




**Supplementary Information**: Record of Transparent Peer Review
