# Supplementary material for: Translational Aspects of DNA Damage Repair in Optimizing Cancer Chemotherapy
Source: Adv Genet (Hoboken). 2025 Dec 22;6(4):e00036. doi: 10.1002/ggn2.202500036 (PMC12747549; doi:10.1002/ggn2.202500036)
Supplement: Supplementary file 1 — Supplementary Information: Record of Transparent Peer Review [file GGN2-6-e00036-s001.docx]

Record of Transparent Peer Review

ggn2.202500036

Translational Aspects of DNA Damage Repair in Optimizing Cancer Chemotherapy

Data submitted: 05-June-2025

1st Editorial Decision: 23-Sept-2025

1^st^ Revision Received: 05-Dec-2025

Accepted: 07-Dec-2025

Please find below our point-to-point responses to the reviewers’ comments, including references to the relevant pages:

[To Professor. Reviewer: 1	1](#_Toc1383164811)

[To Professor. Reviewer: 3	7](#_Toc1383164811)

[To Professor. Reviewer: 5	10](#_Toc1383164811)

To Professor. Reviewer: 1

Comments to the Author

This review article aims to elucidate the function of the DNA damage response (DDR) pathway in

contributing to cancer promotion and therapeutic resistance, a timely and relevant topic given the increasing interest in exploiting synthetic lethality in contexts of DDR defects and therapeutic resistance.

While the manuscript presents a broad overview, it currently lacks the detailed specificity required for a comprehensive review. To significantly enhance its impact and utility for translational research and clinical applications, a more intensive focus on the specific molecular mechanisms is needed.

Key Areas for Improvement

1. Detailed Contribution to Chemotherapeutic Resistance: The manuscript would benefit from a more in-depth discussion of the specific proteins, genes, or biological processes (e.g., phosphorylation, ubiquitination, glycosylation) within each DNA repair pathway that contribute to chemotherapeutic resistance. This could be effectively presented in a summary table, cross-referencing these elements with supporting translational or clinical research and specifying the particular cancer types involved.

Q1-Reply: Thank you for your valuable feedback on our manuscript. We appreciate your thorough review and the opportunity to improve our work.

We agree with the reviewer's suggestion to delve into the elements associated with chemotherapy resistance within each DNA repair pathway, cross-reference them with supporting translational or clinical studies, and specify the cancer types involved. To address this, we have elucidated the pivotal roles of specific proteins, genes, or biological processes that influence DNA repair mechanisms.

We have revised the relevant descriptions within the manuscript as follows:

**2.1 HRR**

Defective DNA damage repair (DDR), driven by endogenous or exogenous factors, predisposes cells to replication stress and represents a critical determinant of cancer cell therapeutic vulnerability. In tumor cells, the assembly of the HR complex facilitates homologous recombination (HR); by modulating kinases to coordinate the DNA damage response (DDR) and suppress cell cycle progression, this mechanism counteracts genotoxic stress and repairs double-strand breaks (DSBs), thereby preserving genomic stability. In the context of the cellular response to replication stress, ATR kinase plays a central role in activating DNA damage tolerance pathways. Impediments to replication fork progression—induced by replication stress or double-strand breaks—result in helicase-polymerase uncoupling and the subsequent generation of single-stranded DNA (ssDNA) at stalled forks. Replication protein A (RPA) coats the ssDNA at stalled forks and recruits ATR via its binding partner, ATR-interacting protein (ATRIP). Upon activation by DNA topoisomerase II binding protein 1 (TopBP1), ATR phosphorylates CHK1 and suppresses cyclin-dependent kinase 2 (CDK2) activity, thereby triggering ATR-CHK1 pathway-mediated S-G2 cell cycle arrest and facilitating the HR process. For instance, in oxaliplatin-based therapy, widespread activation of the HRR mechanism repairs drug-induced DNA damage, promoting the development of resistant phenotypes and significantly reducing chemotherapy efficacy. With the discovery of multiple targetable proteins, including ataxia telangiectasia mutated protein and CHK1/2, HRR and its related therapeutic strategies have emerged as important areas in precision oncology research. Homologous recombination deficiency (HRD) has been established as a predictive biomarker for PARPi therapy in ovarian cancer; furthermore, distinct HRD phenotypes correlate with somatic mutations and the epigenetic silencing of BRCA1 and BRCA2 in breast, prostate, and pancreatic cancers.

**2.2 NHEJ**

Key components of the non-homologous end joining (NHEJ) repair pathway include the Ku heterodimer (comprising Ku70 and Ku80) and the DNA-PK catalytic subunit (DNA-PKcs), which play a pivotal role in recognizing double-strand breaks (DSBs) and activating downstream NHEJ signaling. During the initiation of NHEJ, the Ku heterodimer detects DNA break sites and binds to the broken ends; this binding induces a conformational change that recruits the DNA-PK catalytic subunit, thereby assembling an active DNA-PK complex. Upon assembly, this complex undergoes autophosphorylation and mediates the phosphorylation of the XRCC4-Ligase IV complex, XRCC4-like factor (XLF), and XLF homologs. These events recruit core NHEJ proteins to the damage site to execute ligation, stabilize broken ends against nuclease degradation, and fully activate the NHEJ repair machinery. While this process is crucial for maintaining genomic stability, it also mitigates the cytotoxic effects of DNA-damaging agents—such as ionizing radiation (IR), IR mimetics, and certain alkylating agents—thereby potentially driving tumor progression and conferring cellular drug resistance. Specifically, studies indicate that in non-melanoma skin cancer, the upregulation of Ku70 and Ku80 levels correlates with drug resistance, contributing to a poor prognosis.

**2.3 BER**

AP endonuclease 1 (APE1) hydrolyzes the phosphodiester backbone at the AP site, generating a single-strand break (SSB) characterized by a 3'-hydroxyl group and a 5'-deoxyribose phosphate (5'-dRP) terminus. Subsequently, poly (ADP-ribose) polymerase 1 (PARP1) recognizes the strand break, facilitating DNA end protection via poly-ADP-ribosylation (PARylation) and recruiting downstream effector proteins. Mediated by DNA ligase I or the complexes of DNA ligase III and X-ray repair cross-complementing protein 1 (XRCC1), repair synthesis fills the gap, culminating in the covalent ligation of the nascent nucleotides to the parental DNA strand by DNA ligases.

Consequently, owing to the protective activity of MGMT in malignancies with high expression levels—such as brain, skin, thymus, and liver cancers—significant resistance to exogenous alkylating agents is frequently observed.

**2.4 NER**

The nucleotide excision repair (NER) process comprises two distinct subpathways: global genome NER (GG-NER) and transcription-coupled NER (TC-NER). GG-NER initiates damage recognition via a complex consisting of Xeroderma Pigmentosum Complementation Group C (XPC), HR23B, and Centrin2; upon binding to chemically modified nucleotides, this complex recruits the XPF-ERCC1 endonuclease complex to generate an exposed single-stranded DNA (ssDNA) gap within the double helix. Assisted by PCNA and replication factors, DNA polymerases perform DNA synthesis to fill the ssDNA gap, which is subsequently ligated by DNA ligase. Conversely, the TC-NER pathway is initiated at sites of bulky DNA lesions, where Cockayne Syndrome Protein B (CSB) binds to stalled RNA polymerase and recruits Cockayne Syndrome Protein A (CSA) to induce chromatin remodeling, thereby activating the repair process. The NER system is capable of repairing DNA damage induced by platinum-based agents, a characteristic closely associated with the development of chemotherapy resistance in tumor cells. Mutations in the NER-related gene ERCC2 may compromise cellular repair capacity, potentially promoting the progression of bladder cancer while attenuating cellular susceptibility to clinical therapeutic agents.

**2.5 MMR**

The MMR system utilizes MutS homologs to recognize mismatched nucleotides and activates MutH to cleave the unmethylated DNA strand, thereby transmitting the damage signal to downstream excision repair complexes. Exonucleases excise the nascent strand extending from the cleavage site to the mismatch; subsequently, DNA polymerase III and DNA ligase fill the resulting gap and seal the nick, thereby completing the repair process to maintain genomic integrity.

Aberrant expression of key MMR genes significantly influences predisposition to colorectal and gastric malignancies, as well as the cellular proficiency in repairing chemotherapy-induced DNA damage, ultimately leading to the development of chemotherapy resistance.

**References**

25.	da Costa AABA, Chowdhury D, Shapiro GI, D’Andrea AD, Konstantinopoulos PA. Targeting replication stress in cancer therapy. Nat Rev Drug Discov. 2023 Jan;22(1):38–58.

26.	Tan J, Sun X, Zhao H, Guan H, Gao S, Zhou PK. Double-strand DNA break repair: molecular mechanisms and therapeutic targets. MedComm (2020). 2023 Oct;4(5):e388.

30.	Moon J, Kitty I, Renata K, Qin S, Zhao F, Kim W. DNA Damage and Its Role in Cancer Therapeutics. Int J Mol Sci. 2023 Mar 1;24(5).

31.	Grundy GJ, Parsons JL. Base excision repair and its implications to cancer therapy. Essays Biochem. 2020 Oct 26;64(5):831–43.

32.	Çağlayan M. The ligation of pol β mismatch insertion products governs the formation of promutagenic base excision DNA repair intermediates. Nucleic Acids Res. 2020 Apr 17;48(7):3708–21.

34.	Peng Y, Pei H. DNA alkylation lesion repair: outcomes and implications in cancer chemotherapy. J Zhejiang Univ Sci B. 2021 Jan 15;22(1):47–62.

35.	McPherson KS, Korzhnev DM. Targeting protein-protein interactions in the DNA damage response pathways for cancer chemotherapy. RSC Chem Biol. 2021 Aug 5;2(4):1167–95.

Relevant text has been supplemented in Section 2. This adjustment helps maintain the coherence of the paper while precisely focusing on the detailed contributions of specific proteins, genes, or biological processes to chemotherapy resistance across different DNA repair pathways.

To clearly illustrate this trajectory, we have cross-referenced the core therapeutic elements of DNA repair processes, their mechanisms of action, corresponding key studies, and applicable cancer types in the following table. This summary clearly delineates the translational pathway from target identification and mechanistic exploration to clinical validation.

We have summarized the relevant sections in the manuscript as follows:

| **DDR Pathway** | **Key Proteins/Genes Involved** | **Mechanism of Action in Chemotherapy Response** | **Targeted**  **Cancer**  **Types**  **(Examples)** | **Clinical/Translational Implications** | **Ref.** |
| --- | --- | --- | --- | --- | --- |
| **HRR**  (Homologous Recombination  Repair) | **BRCA1/2,**  ATM,  ATR | **Sensitivity:** Deficiency (HRD) prevents repair of platinum-induced ICLs and DSBs.  **Resistance:**Reversion mutations in BRCA1/2 or upregulation of RAD51 restores HRR function. | Ovarian Cancer, Breast Cancer, Prostate Cancer | Predictive biomarker for Platinum sensitivity and PARP inhibitors  efficacy (e.g., Olaparib);  Guidance for the personalized treatment of solid tumors | [24,  26] |
| **NHEJ** (Non-  Homologous  End Joining) | **DNA-PKcs**, Ku70/80 | **Sensitivity:** The non-homologous end joining (NHEJ) pathway is characterized by its low fidelity, which predisposes cells to genomic instability  **Resistance:** Upregulation of DNA-PKcs/Ku  promotes rapid repair of DSBs induced by alky lating agents and IR | Non-Melanoma Skin Cancer,  Glioblastoma | High Ku70/80 levels correlate with p oor prognosis;  DNA-PK inhibitors sensitize tumo rs to chemotherapy/radiotherapy. | [29,  30] |
| **BER** (Base Excision  Repair) | **PARP1**,  XRCC1,  MGMT | **Sensitivity:** PARP trapping blocks BER, leading to toxic DSBs in S-phase.  **Resistance:** High MGMT levels directly remove alkyl groups (e.g., from temozolomide). | Malignancies of the brain, skin, thymus, and liver | MGMT promoter methylation status predicts Temozolomide response;  PARP inhibitors target BER dependency. | [31,  32] |
| **NER**  (Nucleotide Excision  Repair) | **ERCC1**,  ERCC2 (XPD), XPC | **Sensitivity:** Targeting key enzymes within the nucleotide excision repair (NER) pathway to impede the DNA repair machinery  **Resistance:** Enhanced removal of bulky  platinum-DNA adducts by ERCC1-XPF complex reduces drug cytotoxicity. | Bladder  Cancer | ERCC2 mutations may be associated with cellular repair capacity, thereby driving the progression of bladder cancer and reducing cellular susceptibility | [35,  36] |
| **MMR**  (Mismatch Repair) | **MutS,**  DNA polymerase III,  DNA ligase | **Sensitivity:** dMMR causes high TMB, increasing neoantigens.  **Resistance:** MMR deficiency leads to tolerance of alkylating agent-induced mispairs | Colorectal and gastric malignancies | The deletion or inactivation of mismatch repair-related genes has the potential to modulate the tumor microenvironment | [38,  39] |

We believe these modifications enhance the persuasiveness of the paper by ensuring that all discussed processes are directly linked to chemotherapy resistance, thereby making our review more lucid and accurate.

2. Current Gaps and Future Directions: To provide clearer guidance for the field, the review should more explicitly identify the current gaps in our understanding of the DDR pathway's role in cancer and therapeutic resistance. Additionally, a more detailed outline of future directions for translational research and clinical application would be highly beneficial. This would help clinicians and researchers identify promising avenues for further investigation and therapeutic development.

Q2-Reply: We appreciate the reviewer's meticulous attention to the knowledge gaps concerning the role of DDR pathways in cancer and therapeutic resistance, and we acknowledge the need for further clarification. You correctly pointed out the necessity of providing a detailed overview of future directions for translational research and clinical applications. This will aid clinicians and researchers in identifying promising avenues for further investigation and therapeutic development.

We have discussed preclinical approaches for selective drug delivery aimed at addressing chemotherapy toxicity and overcoming drug resistance. The results demonstrate that these agents exhibit improved safety profiles compared to traditional drugs, which holds significant implications.

In the current manuscript, we have revised the relevant descriptions as follows:

However, existing DDRi generally suffer from issues such as insufficient selectivity and systemic toxicity, commonly causing adverse events such as diarrhea, fatigue, nausea, neutropenia, and leukopenia. Mechanisms including the restoration of BRCA1/2 function, stabilization of replication forks, and drug target-related effects frequently drive acquired resistance, limiting their clinical application. Therefore, developing next-generation DDR-targeted drugs with higher selectivity and lower toxicity is of significant importance for enhancing clinical therapeutic outcomes. To mitigate chemotherapy-associated toxicity and develop DDR strategies capable of overcoming resistance, a key approach involves the selective delivery of chemotherapeutic agents. For instance, CRLX101 uses a nanoparticle formulation for preferential delivery to tumors via leaky vasculature, demonstrating an improved safety profile compared to conventional Top1 inhibitors such as topotecan and irinotecan. Continued investigation into the antitumor efficacy and tolerability of combining chemotherapeutic agents with DDR inhibitors is of significant clinical importance. Other potential approaches to address therapeutic challenges associated with novel DDR inhibitors include implementing combination therapies to potentiate antitumor effects and inhibiting mutator phenotypes to delay resistance onset.

**References**

95.	O’Connor MJ. Targeting the DNA Damage Response in Cancer. Mol Cell. 2015 Nov 19;60(4):547–60.

**To Professor. Reviewer: 3**

Major Comments

1. The description of DDR's dual role in the introduction section contains logical gaps. The statement "DDR exhibits a dual role in tumor occurrence, development, and treatment" requires clearer transitions. It is recommended to provide a clear explanation of how DDR transforms from its protective role to mechanisms that promote tumorigenesis after describing its protective functions.

Q1-Reply: We thank the reviewer for the valuable suggestion to explicitly describe the impact of the dual role of DDR, and specifically to provide a clear explanation of the mechanisms governing how DDR transitions from a protective function to promoting tumorigenesis.

We have supplemented the section "DDR exhibits a dual role in tumorigenesis, progression, and treatment" with details regarding the transition of DDR's protective effects, explicitly clarifying the influence of critical processes—such as germline functional alterations in DDR genes, epigenetic silencing, and modulation of the tumor microenvironment—on DDR-driven tumor development.

In the manuscript, we have revised the relevant content as follows:

DDR exhibits a dual role in tumor occurrence, development, and treatment: on one hand, during the normal cell cycle, the timely repair of DNA damage is indispensable for maintaining genomic stability, thereby averting cellular mutations and tumorigenesis; conversely, the DDR system may facilitate the emergence of chemoresistance by repairing DNA lesions induced by chemotherapeutic agents. Evidence suggests that the survival of damaged cells correlates with germline functional alterations in DDR genes; mechanisms including epigenetic silencing and tumor microenvironment modulation enhance DDR pathway activity, thereby repurposing the protective role of DDR into a critical driver of tumor progression that enables cancer cell tolerance to DNA damage and adaptation to chemotherapy.

**References**

15.	Gueble SE, Bindra RS. Oncometabolites as Regulators of DNA Damage Response and Repair. Semin Radiat Oncol. 2022 Jan;32(1):82–94.

These revisions delineate the boundary between DDR's role in preventing cellular mutations and its contribution to chemotherapy resistance, enabling readers to gain a deeper understanding of the unique characteristics of this dual role and the clinical significance of investigating DNA damage mechanisms.

2. The legend description for Figure 1 is overly lengthy and should be simplified while highlighting the core mechanisms.

Q2-Reply: We have simplified the legend description for Figure 1 to highlight the core mechanisms.

In the revised manuscript, we have completed the revision of the description:

I. Platinum-based therapies induce DNA inter-strand crosslinks (ICLs), which exacerbate genomic instability and impede DNA replication. In BRCA1/2-deficient tumor cells, the repair of these DNA crosslinks—which relies strictly on the BRCA1/2-mediated homologous recombination repair (HRR) pathway—is compromised, ultimately triggering cell cycle arrest.

II. Alkylating antitumor agents induce DNA alkylation modifications, interfering with DNA repair processes and triggering base mismatches. MMR functional deficiencies lead to ineffective DNA error correction, subsequently inducing MSI and significantly increasing single-nucleotide substitution frequency, thereby suppressing the emergence of chemotherapy resistance.

III.Dysfunction of the ATM/ATR signaling pathways impairs the activation of both ATM-mediated homologous recombination repair (HRR) and non-homologous end joining (NHEJ); this results in the accumulation of DNA double-strand breaks (DSBs) within tumor cells, ultimately potentiating chemotherapy cytotoxicity.

IV. Mutations in DNA damage repair (DDR) genes serve as valuable molecular biomarkers in oncological research. For instance, the HRDetect system predicts therapeutic responses to platinum-based drugs by detecting homologous recombination repair (HRR) deficiencies stemming from BRCA1/2 mutations; similarly, alterations in the RAD51 gene can be used to evaluate the chemosensitivity of liver cancer cells.

3. In Section 5.2, the statement "selective small molecule inhibitors targeting ATM/ATR have become an important strategy" is too absolute. It is suggested to revise it to "have emerged as a promising strategy" since these inhibitors are still in clinical trial phases.

Q3-Reply: We appreciate your constructive comments on our manuscript. We are particularly grateful for your suggestion to modify the phrase "selective small-molecule inhibitors targeting ATM/ATR have become an important strategy" to "have become a promising strategy" in Section 5.2.

Given that these inhibitors are currently undergoing clinical trials, we have revised the overly absolute statement in Section 5.2:

To overcome this clinical challenge, selective small molecule inhibitors targeting ATM/ATR have become a promising avenue in antitumor drug development and have demonstrated significant clinical potential.

4. The title of Section 6.4 in the future perspectives section, "Exploration of Dynamic Evolution Mechanisms," is not sufficiently clear. It is recommended to revise it to "Understanding the Dynamic Evolution of DDR-Mediated Resistance."

Q4-Reply: We appreciate your valuable feedback regarding the "Future Perspectives" section of our manuscript. We concur with the reviewer's observation that the heading of Section 6.4, "Exploration of dynamic evolutionary mechanisms," was insufficiently clear.

To enhance the relevance of the manuscript, we have revised this section in accordance with your suggestions:

**6.4 Elucidating the Dynamic Evolution of DDR-Mediated Drug Resistance**

**To Professor. Reviewer: 5**

Comments to the Author

1. While the review covers the five core DDR pathways involved in chemotherapy resistance, the discussion is somewhat general at present. I recommend incorporating more concrete examples to strengthen the analysis and enhance its translational relevance. The addition of supporting figures or schematics would also help illustrate the key mechanisms and make the content more accessible to readers.

Q1-Reply: We sincerely appreciate the reviewer's insightful suggestion aimed at enhancing the analytical depth and translational relevance of our manuscript. You are correct that incorporating specific examples to strengthen the analysis of key mechanisms within the five core DDR pathways, along with adding supporting figures or schematics to facilitate reader comprehension, is crucial for elucidating the clinical significance of DDR pathways in chemotherapy resistance.

In response to this suggestion, we have thoroughly revisited Section 2 and revised it by explicitly highlighting and expanding upon the specific clinically relevant examples already included in the discussion. The specific improvements are as follows:

In Section 2.1, we emphasized the role of HRR hyperactivation in conferring oxaliplatin resistance and detailed how BRCA1/2 deficiency serves as a well-recognized predictive biomarker for sensitivity to PARP inhibitors in cancers such as ovarian, breast, and prostate cancer.

In Section 2.2, we strengthened the analysis of the association between Ku70 and Ku80 proteins and poor prognosis in non-melanoma skin cancer.

In Section 2.3, we explained the critical role of the DNA repair enzyme MGMT in repairing alkylation damage in brain, skin, thymic, and liver cancers, thereby promoting significant drug resistance.

In Section 2.4, we elucidated the clinical impact of mutations in the NER pathway-related gene ERCC2, noting that these mutations impair the NER pathway, affecting prognosis and cellular susceptibility in cancers such as bladder cancer.

In Section 2.5, we reinforced the profound impact of aberrant expression of key genes in the MMR pathway on susceptibility in colorectal and gastric malignancies.

In the current manuscript, we have completed the revisions to the descriptive sections:

**2.1 HRR**

Defective DNA damage repair (DDR), driven by endogenous or exogenous factors, predisposes cells to replication stress and represents a critical determinant of cancer cell therapeutic vulnerability. In tumor cells, the assembly of the HR complex facilitates homologous recombination (HR); by modulating kinases to coordinate the DNA damage response (DDR) and suppress cell cycle progression, this mechanism counteracts genotoxic stress and repairs double-strand breaks (DSBs), thereby preserving genomic stability. In the context of the cellular response to replication stress, ATR kinase plays a central role in activating DNA damage tolerance pathways. Impediments to replication fork progression—induced by replication stress or double-strand breaks—result in helicase-polymerase uncoupling and the subsequent generation of single-stranded DNA (ssDNA) at stalled forks. Replication protein A (RPA) coats the ssDNA at stalled forks and recruits ATR via its binding partner, ATR-interacting protein (ATRIP). Upon activation by DNA topoisomerase II binding protein 1 (TopBP1), ATR phosphorylates CHK1 and suppresses cyclin-dependent kinase 2 (CDK2) activity, thereby triggering ATR-CHK1 pathway-mediated S-G2 cell cycle arrest and facilitating the HR process. For instance, in oxaliplatin-based therapy, widespread activation of the HRR mechanism repairs drug-induced DNA damage, promoting the development of resistant phenotypes and significantly reducing chemotherapy efficacy. With the discovery of multiple targetable proteins, including ataxia telangiectasia mutated protein and CHK1/2, HRR and its related therapeutic strategies have emerged as important areas in precision oncology research. Homologous recombination deficiency (HRD) has been established as a predictive biomarker for PARPi therapy in ovarian cancer; furthermore, distinct HRD phenotypes correlate with somatic mutations and the epigenetic silencing of BRCA1 and BRCA2 in breast, prostate, and pancreatic cancers.

**2.2 NHEJ**

Key components of the non-homologous end joining (NHEJ) repair pathway include the Ku heterodimer (comprising Ku70 and Ku80) and the DNA-PK catalytic subunit (DNA-PKcs), which play a pivotal role in recognizing double-strand breaks (DSBs) and activating downstream NHEJ signaling. During the initiation of NHEJ, the Ku heterodimer detects DNA break sites and binds to the broken ends; this binding induces a conformational change that recruits the DNA-PK catalytic subunit, thereby assembling an active DNA-PK complex. Upon assembly, this complex undergoes autophosphorylation and mediates the phosphorylation of the XRCC4-Ligase IV complex, XRCC4-like factor (XLF), and XLF homologs. These events recruit core NHEJ proteins to the damage site to execute ligation, stabilize broken ends against nuclease degradation, and fully activate the NHEJ repair machinery. While this process is crucial for maintaining genomic stability, it also mitigates the cytotoxic effects of DNA-damaging agents—such as ionizing radiation (IR), IR mimetics, and certain alkylating agents—thereby potentially driving tumor progression and conferring cellular drug resistance. Specifically, studies indicate that in non-melanoma skin cancer, the upregulation of Ku70 and Ku80 levels correlates with drug resistance, contributing to a poor prognosis.

**2.3 BER**

AP endonuclease 1 (APE1) hydrolyzes the phosphodiester backbone at the AP site, generating a single-strand break (SSB) characterized by a 3'-hydroxyl group and a 5'-deoxyribose phosphate (5'-dRP) terminus. Subsequently, poly (ADP-ribose) polymerase 1 (PARP1) recognizes the strand break, facilitating DNA end protection via poly-ADP-ribosylation (PARylation) and recruiting downstream effector proteins. Mediated by DNA ligase I or the complexes of DNA ligase III and X-ray repair cross-complementing protein 1 (XRCC1), repair synthesis fills the gap, culminating in the covalent ligation of the nascent nucleotides to the parental DNA strand by DNA ligases.

Consequently, owing to the protective activity of MGMT in malignancies with high expression levels—such as brain, skin, thymus, and liver cancers—significant resistance to exogenous alkylating agents is frequently observed.

**2.4 NER**

The nucleotide excision repair (NER) process comprises two distinct subpathways: global genome NER (GG-NER) and transcription-coupled NER (TC-NER). GG-NER initiates damage recognition via a complex consisting of Xeroderma Pigmentosum Complementation Group C (XPC), HR23B, and Centrin2; upon binding to chemically modified nucleotides, this complex recruits the XPF-ERCC1 endonuclease complex to generate an exposed single-stranded DNA (ssDNA) gap within the double helix. Assisted by PCNA and replication factors, DNA polymerases perform DNA synthesis to fill the ssDNA gap, which is subsequently ligated by DNA ligase. Conversely, the TC-NER pathway is initiated at sites of bulky DNA lesions, where Cockayne Syndrome Protein B (CSB) binds to stalled RNA polymerase and recruits Cockayne Syndrome Protein A (CSA) to induce chromatin remodeling, thereby activating the repair process. The NER system is capable of repairing DNA damage induced by platinum-based agents, a characteristic closely associated with the development of chemotherapy resistance in tumor cells. Mutations in the NER-related gene ERCC2 may compromise cellular repair capacity, potentially promoting the progression of bladder cancer while attenuating cellular susceptibility to clinical therapeutic agents.

**2.5 MMR**

The MMR system utilizes MutS homologs to recognize mismatched nucleotides and activates MutH to cleave the unmethylated DNA strand, thereby transmitting the damage signal to downstream excision repair complexes. Exonucleases excise the nascent strand extending from the cleavage site to the mismatch; subsequently, DNA polymerase III and DNA ligase fill the resulting gap and seal the nick, thereby completing the repair process to maintain genomic integrity.

Aberrant expression of key MMR genes significantly influences predisposition to colorectal and gastric malignancies, as well as the cellular proficiency in repairing chemotherapy-induced DNA damage, ultimately leading to the development of chemotherapy resistance.

**References**

25.	da Costa AABA, Chowdhury D, Shapiro GI, D’Andrea AD, Konstantinopoulos PA. Targeting replication stress in cancer therapy. Nat Rev Drug Discov. 2023 Jan;22(1):38–58.

26.	Tan J, Sun X, Zhao H, Guan H, Gao S, Zhou PK. Double-strand DNA break repair: molecular mechanisms and therapeutic targets. MedComm (2020). 2023 Oct;4(5):e388.

30.	Moon J, Kitty I, Renata K, Qin S, Zhao F, Kim W. DNA Damage and Its Role in Cancer Therapeutics. Int J Mol Sci. 2023 Mar 1;24(5).

31.	Grundy GJ, Parsons JL. Base excision repair and its implications to cancer therapy. Essays Biochem. 2020 Oct 26;64(5):831–43.

32.	Çağlayan M. The ligation of pol β mismatch insertion products governs the formation of promutagenic base excision DNA repair intermediates. Nucleic Acids Res. 2020 Apr 17;48(7):3708–21.

34.	Peng Y, Pei H. DNA alkylation lesion repair: outcomes and implications in cancer chemotherapy. J Zhejiang Univ Sci B. 2021 Jan 15;22(1):47–62.

35.	McPherson KS, Korzhnev DM. Targeting protein-protein interactions in the DNA damage response pathways for cancer chemotherapy. RSC Chem Biol. 2021 Aug 5;2(4):1167–95.

Regarding the suggestion for supporting schematic diagrams, we have given this careful consideration. Our strategic approach to visual presentation is to move beyond merely illustrating the well-documented canonical pathways themselves; instead, we have dedicated the figures to synthesizing the higher-order integrative concepts that constitute the core contribution of our review. We have developed three summary figures that directly encapsulate and visualize the key mechanisms discussed in Section 2:

Figure 1 synthesizes how defects in specific DDR pathways discussed in Section 2 mechanistically lead to increased tumor susceptibility.

Figure 2 illustrates how the aberrant activation of these same pathways leads to the development of chemotherapy resistance.

Figure 3 outlines future research approaches built upon this foundational knowledge.

These summary figures effectively capture the key mechanisms mentioned by the reviewer and enhance the understanding of the clinical mechanisms of DDR pathways.

We hope these revisions satisfactorily address the reviewer's concerns. We are willing to make any additional modifications if necessary.

2. Sections 1 and 2 currently lack figures. Including appropriate visual representations in these sections would improve readability and aid in conveying complex concepts more effectively

Q2-Reply: We appreciate the reviewer's valuable suggestions regarding the content of Sections 1 and 2. We fully concur that incorporating appropriate visual elements into the review helps enhance content readability and clarity of concept transmission. In response to this comment, we wish to emphasize that Figure 1 was specifically designed to provide a systematic and intuitive integration and summary of the core concepts and mechanisms elucidated in Sections 1 and 2. Figure 1 echoes the central arguments presented in Section 1—specifically, the dual role of the DDR system in maintaining genomic stability and mediating chemotherapeutic responses, as well as how the status of key DDR molecules serves as a biomarker for predicting chemotherapy efficacy. Concurrently, Figure 1 encompasses the critical roles of the five major DDR core pathways detailed in Section 2 regarding chemotherapy response, further linking functional deficiencies or abnormalities in these pathways (e.g., BRCA1/2 deficiency, MMR defects, and ATM/ATR signaling anomalies) to the resulting alterations in chemosensitivity. Therefore, rather than adding redundant diagrams targeting specific pathways, we prioritized this comprehensive figure to capture the core causal relationships central to the translational focus of this review. We believe that this illustration effectively enhances the reader's comprehension of the complex concepts introduced in the initial sections.

3. The manuscript contains a large number of abbreviations, which may reduce accessibility for general readers. I suggest minimizing unnecessary abbreviations and ensuring that all essential terms are clearly defined upon first use. A table summarizing frequently used abbreviations could also help improve clarity

Q3-Reply: We agree with the reviewer's observation that the manuscript contains a significant number of abbreviations; upon re-examination, we have minimized unnecessary abbreviations, clearly defined all essential terms upon their first mention, and included a table summarizing common abbreviations to enhance accessibility for readers.

In this manuscript, we have completed the revisions to the descriptive sections as follows:

**ABBREVIATONS**

***DDR:*** DNA Damage Repair

***SSBs:*** Single Strand Breaks

***DSBs:*** Double-Strand Breaks

***ICLs:*** inter-strand crosslinks

***ATM:*** Ataxia Telangiectasia Mutated

***ATR:*** Ataxia telangiectasia and Rad3-related

***PARP:*** Poly ADP-ribose polymerase

***PARPi:*** PARP inhibitors

***DDRi:*** DNA Damage Response inhibitors

***HRR:*** Homologous Recombination Repair

***NHEJ:*** Non-Homologous End Joining

***BER:*** Base Excision Repair

***NER:*** Nucleotide Excision Repair

***MMR:*** Mismatch Repair

***CHK1/2:*** Checkpoint Kinase1/2

***HR:*** Homologous Recombination

***HRRm:*** HRR gene mutation

***DNA-PK:*** DNA-dependent protein kinase

***DNA-PKcs:*** DNA-dependent protein kinase catalytic subunit

***HRD:*** Homologous Recombination deficient

***TME:*** Tumor Microenvironment

***MSI:*** microsatellite instability

***TMB:*** tumor mutational burden

***NSCLC:*** non-small cell lung cancer

***CSCs:*** cancer stem cells

***PGE2:*** Prostaglandin E2

***PTM:*** Post-translational modification

***ESC:*** embryonic stem cell

***PROTAC:*** Proteolysis Targeting Chimera

***LncRNAs:*** Long Non-Coding RNAs

***MiRNAs:*** MicroRNAs

***CircRNA:*** Circular RNA

***ICB:*** immune checkpoint blockade

4. Section 4 provides a good overview of DDR-mediated resistance mechanisms, but the role of cancer stem cells and microenvironmental interactions could be further substantiated with examples or recent references. Similarly, Section 5 on DDR inhibitors would benefit from a more detailed discussion of representative agents (e.g., PARP, ATR, CHK1 inhibitors), their mechanisms, and progress in clinical trials.

Q4-Reply: We thank the reviewer for the detailed attention to the section on DDR-mediated drug resistance mechanisms; you noted the need to substantiate the interactions between cancer stem cells (CSCs) and the microenvironment in detail, and to discuss the mechanisms and clinical trial progress of representative DDR inhibitors in depth. We agree with this insightful point.

In response to this suggestion, we have revised Sections 4 and 5. The specific improvements are as follows:

We provided an in-depth analysis of how hypoxia regulation, angiogenic dysregulation, and distinct niches interact with CSCs to contribute to therapeutic failure.

We highlighted the signaling pathways by which CSCs evade immune surveillance via exosomes.

We expanded the content regarding "trapping" theory to clearly differentiate the therapeutic strategies involving potent inhibitors versus allosteric inhibitors.

We discussed the current progress of clinical trials for highly selective ATR inhibitors, specifically focusing on the efficacy of Berzosertib in inhibiting pancreatic tumor progression and the safety profile of WSD0628 in the treatment of persistent brain tumors.

We emphasized the mechanism underlying the combination of the CHK1/2 inhibitor prexasertib with cisplatin, as well as its impact on head and neck squamous cell carcinoma (HNSCC).

We clarified the therapeutic feasibility of the DNA-PK inhibitor CC-115 in patients with brain tumors and various other malignancies.

In the manuscript, we have completed the revisions to the descriptive sections as follows:

**5.1 PARPi**

The PARP trapping theory postulates that PARP inhibitors competitively bind to the NAD+-binding domain of PARP1, thereby inhibiting its catalytic activity. Consequently, prevented from undergoing auto-PARylation, PARP1 becomes entrapped on DNA, causing stalled replication forks and double-strand breaks (DSBs) at damage sites as cells progress through the S phase. In HR-deficient tumor cells, where DSB repair relies on compensatory non-homologous end joining (NHEJ), genomic instability is exacerbated, driving the synthetic lethality associated with BRCA1 deficiency and PARP inhibition. (2) conversely, allosteric inhibitors, exemplified by veliparib, induce conformational changes in PARP1 that attenuate DNA binding and promote its dissociation from DNA. Furthermore, through structure-guided optimization, allosteric inhibitors can be engineered to shift from promoting release to enhancing retention, thereby conferring potent PARP1 trapping potency and increased cytotoxicity against cancer cells.

**5.2 ATM/ATR Inhibitors**

To overcome this clinical challenge, selective small molecule inhibitors targeting ATM/ATR have become a promising avenue in antitumor drug development and have demonstrated significant clinical potential. ATM/ATR inhibitors specifically target key kinases in the DDR pathway, thereby interfering with the cell cycle checkpoint regulatory network in tumor cells. Among these, the natural compound Shikonin specifically induces proteasome-dependent degradation of ATM-ATR Interacting Protein (ATRIP), inhibiting the activation of upstream regulatory factors in the DDR signaling pathway, thereby enhancing the sensitivity of tumor cells to chemotherapeutic agents. Research indicates that the combined application of ATR inhibitors and DNA damage inducers triggers synthetic lethal effects, significantly enhancing the antitumor activity of chemotherapeutic agents such as cisplatin and the PARPi veliparib, the highly selective ATR inhibitor Berzosertib has demonstrated efficacy in suppressing pancreatic tumor progression while exhibiting minimal off-target toxicity in normal tissues. Preliminary data from Phase I trials involving advanced solid tumors suggest that combining Berzosertib with the platinum-based agent Cisplatin exhibits a favorable safety and tolerability profile. Current clinical trials are evaluating the safety, pharmacokinetics, and preliminary antitumor efficacy of the potent ATM inhibitor WSD0628 in treating persistent brain tumors, thus providing new therapeutic approaches for optimizing clinical combination drug strategies.

**5.3 CHK1/2 Inhibitors**

Preclinical studies have investigated the efficacy of the CHK1/2 inhibitor prexasertib combined with cisplatin against head and neck squamous cell carcinoma. Results indicated that prexasertib abrogated chemotherapy-induced checkpoint activation and modulated cell cycle distribution, resulting in sustained DNA damage; these findings highlight the potential for enhanced clinical benefits and offer a rationale for developing novel therapeutic strategies.

**5.4 DNA-PK Inhibitors**

A preliminary clinical trial demonstrated the therapeutic efficacy of the DNA-PK inhibitor CC-115 in patients with brain tumors and diverse malignancies, positioning it as a promising candidate for future anticancer interventions. However, owing to modest efficacy or significant toxicity, certain inhibitors, such as XL765, have exhibited limited utility in clinical trials and remain unsuitable for clinical application.

**References**

67.	Rose M, Burgess JT, O’Byrne K, Richard DJ, Bolderson E. PARP Inhibitors: Clinical Relevance, Mechanisms of Action and Tumor Resistance. Front Cell Dev Biol. 2020;8:564601.

68.	Miramova A, Gartner A, Ivanov D. How to sensitize glioblastomas to temozolomide chemotherapy: a gap-centered view. Front Cell Dev Biol. 2024;12:1436563.

69.	Zandarashvili L, Langelier MF, Velagapudi UK, Hancock MA, Steffen JD, Billur R, et al. Structural basis for allosteric PARP-1 retention on DNA breaks. Science. 2020 Apr 3;368(6486).

These revisions provide a more accurate reflection of the comprehensive landscape involving CSCs and the microenvironment, while simultaneously analyzing the mechanisms of action and clinical trial progress of representative DDR inhibitors.

5. The outlook in Section 6 is well-structured and forward-looking. However, the review would be strengthened by explicitly highlighting ongoing challenges (e.g., toxicity of DDR inhibitors, acquired resistance to DDRi, patient selection based on biomarkers) and possible strategies to overcome them. This would provide a more balanced perspective on the opportunities and limitations in the field.

Q5-Reply: We appreciate your constructive comments on our manuscript. Your suggestion to explicitly emphasize persistent challenges and strategies for overcoming them provides a more balanced perspective on the opportunities and limitations within this field.

We have expanded the discussion on current research limitations in Section 6 to provide a deeper insight into the clinical challenges that remain to be addressed.

We have examined critical data pending analysis in clinical trials, evaluated CRISPR functional genomic screening technologies based on the concept of synthetic lethality, and clarified the significance of biomarkers in precisely selecting therapeutic regimens tailored to individual patient characteristics.

Regarding recent advances in acquired resistance to DDR inhibitors (DDRi), we have addressed the most common adverse events, key processes prone to inducing acquired resistance, and other potential approaches to resolve therapeutic issues associated with novel DDR inhibitors.

In investigating the clinical application potential of emerging DDR PROTAC therapies, we have expanded the clarification regarding the biological functions and regulatory mechanisms of potential targets to underscore their significant clinical value.

We have highlighted issues in multiple current clinical trials wherein the safety and efficacy of combining DNA repair agents with immune checkpoint blockade are influenced by factors such as dosage and the therapeutic context.

We have incorporated these descriptive revisions into the manuscript:

**6 Future Perspectives**

Although research into DNA damage repair (DDR) is ushering in a new era of precision medicine, current progress remains constrained by several limitations: the molecular heterogeneity of tumor DDR remains to be fully elucidated, the toxicity profiles of DDR inhibitors have not been completely resolved, the safety of synergistic treatment paradigms requires rigorous exploration, and the interplay between DDR gene mutations and the tumor immune microenvironment remains ill-defined. To address these challenges, future research and clinical translation should prioritize the implementation of personalized chemotherapy strategies through systematic DDR pathway profiling, the continued development of novel DDR inhibitors to reverse chemotherapy resistance, the comprehensive investigation of synergistic paradigms involving DDR, immunotherapy, and radiotherapy, and the systematic elucidation of the dynamic evolutionary mechanisms by which DDR modulates chemotherapy resistance.

**6.1 Personalized Chemotherapy Strategies Based on Precise DDR Classification**

For instance, the predictive utility of HRD, a currently widely utilized biomarker, may be compromised by resistance mechanisms arising from prior treatments. To address these challenges, researchers are actively exploring innovative therapeutic strategies and technical platforms to elucidate the association between DDR gene polymorphisms and therapeutic responses. Clinical trials are continuously evaluating key predictive factors—such as genomic scar scores, BRCA1/2 promoter methylation, and RAD51 loading—while analyzing critical data regarding somatic status, allele-specific copy numbers, and germline characteristics, thereby achieving dynamic monitoring of HRD function. This approach may profoundly refine biomarker correlations and facilitate the construction of a multi-dimensional biomarker-based assessment system for DDR pathway function, ultimately maximizing the efficacy of individualized treatment strategies. Given the limitations imposed by primary and acquired drug resistance, the therapeutic efficacy and durability of clinical drug combinations often fail to realize truly personalized treatment strategies; therefore, the development of technologies, such as the high-sensitivity detection of DDR defects, is required to precisely screen combination regimens tailored to individual patient profiles, thereby enhancing treatment precision. CRISPR functional genomic screening technology, based on the concept of synthetic lethality, enables the comprehensive functional genomic assessment of all known cancer genes, facilitating the systematic screening of synthetic lethal drug targets in human cancer. Through anchor screens, the CRISPR system can be applied to identify drug combinations involving novel targets, providing a promising pathway for establishing the biomarker context for patient selection in clinical trial development.

**6.2 Development of Novel DDRi to Overcome Chemoresistance**

However, existing DDRi generally suffer from issues such as insufficient selectivity and systemic toxicity, commonly causing adverse events such as diarrhea, fatigue, nausea, neutropenia, and leukopenia. Mechanisms including the restoration of BRCA1/2 function, stabilization of replication forks, and drug target-related effects frequently drive acquired resistance, limiting their clinical application. Therefore, developing next-generation DDR-targeted drugs with higher selectivity and lower toxicity is of significant importance for enhancing clinical therapeutic outcomes. To mitigate chemotherapy-associated toxicity and develop DDR strategies capable of overcoming resistance, a key approach involves the selective delivery of chemotherapeutic agents. For instance, CRLX101 uses a nanoparticle formulation for preferential delivery to tumors via leaky vasculature, demonstrating an improved safety profile compared to conventional Top1 inhibitors such as topotecan and irinotecan. Continued investigation into the antitumor efficacy and tolerability of combining chemotherapeutic agents with DDR inhibitors is of significant clinical importance. Other potential approaches to address therapeutic challenges associated with novel DDR inhibitors include implementing combination therapies to potentiate antitumor effects and inhibiting mutator phenotypes to delay resistance onset.

**6.3 Paradigms of Synergistic Combination Therapy with DDR and Chemotherapy**

By facilitating the degradation of target proteins, DDR-targeting PROTACs can circumvent resistance mechanisms arising from mutations or alternative signaling pathways, thereby imparting enhanced selectivity to traditional DDR inhibitors. To promote the clinical translation of PROTAC technology, it is imperative to thoroughly elucidate the biological functions and regulatory mechanisms of potential targets, evaluate the off-target effects and toxicities associated with targeting specific DDR proteins, and maximize the clinical potential of these emerging therapeutic modalities.

**6.4 Elucidating the Dynamic Evolution of DDR-Mediated Drug Resistance**

Numerous ongoing clinical trials are evaluating the safety and efficacy of combining DNA repair agents with immune checkpoint blockade across diverse dosing and scheduling regimens, seeking to address the context-dependent variability in optimal timing.

**References**

92. Huang A, Garraway LA, Ashworth A, Weber B. Synthetic lethality as an engine for cancer drug target discovery. Nat Rev Drug Discov. 2020 Jan;19(1):23–38.

95.	O’Connor MJ. Targeting the DNA Damage Response in Cancer. Mol Cell. 2015 Nov 19;60(4):547–60.

98.	Cheng B, Fei X, Ding Z, Peng X, Su Z, Pan W, et al. Recent Progress in DNA Damage Response-Targeting PROTAC Degraders. J Med Chem. 2024 May 9;67(9):6906–21.

We tried our best to improve the manuscript and made some changes in the manuscript.

We appreciate for Editors/Reviewers’ warm work earnestly, and hope that the correction will meet with approval.

Once again, thank you very much for your comments and suggestions.
